# Evaluating the pollution footprint of traffic-derived trace elements in roadside soil and the associated risks in a non-metropolitan city

**DOI:** 10.1007/s10661-026-15727-x

**Published:** 2026-08-06

**Authors:** Sutapa Adhikari

**Affiliations:** 1 Present Address: Unit for Environmental Sciences and Management, North-West University, Potchefstroom, 2531 South Africa; 2 South African Environmental Observation Network, Ndlovu Node, WITS Rural Campus, Acornhoek, 1360 South Africa

**Keywords:** Arsenic, Emerging contaminant, Risk assessment, Soil pollution, Traffic, Urban

## Abstract

This study evaluated the influence of multiple traffic-related conditions, including proximity to a road, car parking, highway, road junction, and transport stop, commonly observed yet largely overlooked in many non-metropolitan cities of developing nations, on trace element (TE) contamination in roadside soil and the associated risks in a secondary South African city. Among the evaluated TEs (As, Ba, Cd, Co, Cr, Cu, Mn, Ni, Pb, Sb, Zn), mean Cu and Pb concentrations exceeded national soil quality guidelines at 100% and 86% of sites, respectively, whereas pollution assessment revealed generally moderate Sb, Pb, Cr, Ni, Co, Mn, and Zn contamination at > 71% of sites; indicating 73% of TEs as potential environmental threats. Of the seven examined localities, a mean pollution load index above 1 suggested soil pollution at a site affected by most traffic conditions. Regression analysis indicated positive relationships between road proximity and concentrations of all TEs, with significant (*p* < 0.05) associations for 75% of the identified contaminants, along with As and Ba. Significant positive correlations between TEs exhibiting higher contamination suggested shared sources. This was further supported by principal component analysis, which associated 73% of TEs with one or more traffic variables, primarily road proximity, transport stop, and highway, and secondarily car parking. Moreover, origin from multiple traffic sources was suggested for most TEs. Nearly moderate ecological risks for Cd and Pb at two sampling points, and potential cancer risks for children from As, require follow-up investigation. The findings provide valuable insights into the globally underexamined pollution footprint of multiple traffic-related factors and highlight the need for their management to promote ecosystem sustainability in comparable cities.

**Supplementary Information:**

The online version contains supplementary material available at https://doi.org/10.1007/s10661-026-15727-x.

## Introduction

Soil represents one of the most susceptible environmental matrices to anthropogenic pollution, and over decades, trace elements (TEs) have emerged as persistent and ubiquitous contaminants in urban soils (Li et al., 2018; Sager, 2020; Tiller, 1992). Since soil pollution has a far-reaching impact on the environment and its biological component, this matrix has been used as a critical indicator of human influence on urban ecosystems (Ajmone-Marsan & Biasioli, 2010; Carrero et al., 2013; Grzebisz et al., 2002; Tiller, 1992). Evaluating soil pollution and related ecological and human health risks is hence essential to inform pollution monitoring, regulatory frameworks, and management and remediation strategies. In the long run, such interventions contribute to achieving the United Nations Sustainable Development Goals (SDGs), particularly SDG 3 (Good Health and Well-Being) and SDG 11 (Sustainable Cities and Communities), which remain challenging for many urban areas worldwide, especially relatively smaller but growing cities in developing nations.

While diverse sources contribute to TE pollution of urban edaphic systems, traffic-related processes, including automobile emissions and the degradation of road, pavement, and other infrastructure materials, remain a key contributor, largely driven by the continuous expansion of road networks and the increasing number of vehicles in cities across the globe (Ajmone-Marsan & Biasioli, 2010; Fussell et al., 2022; Hjortenkrans et al., 2006; Pan et al., 2017; Thorpe & Harrison, 2008; Werkenthin et al., 2014). Traffic factors release a broad spectrum of TEs with differing hazard potentials, including more toxic arsenic (As), cadmium (Cd), and lead (Pb), as well as relatively less hazardous barium (Ba), cobalt (Co), chromium (Cr), copper (Cu), manganese (Mn), nickel (Ni), and zinc (Zn) (Carver & Gallicchio, 2017; Miletić et al., 2023). Compared with these well-studied TEs, the emerging contaminant antimony (Sb), despite its extensive use in automobile components (Bukowiecki et al., 2009; Dousova et al., 2020; Fort et al., 2016; Richardson, 2010), remains underinvestigated in roadside environments in developing nations. Though the health impacts of Sb are still being investigated, existing evidence indicates potential adverse effects on human health, with some vehicular activity generated formations classified as probable human carcinogens (Fort et al., 2016). Overall, traffic emissions constitute a substantial proportion of the atmospheric pollution profile (Carrero et al., 2013). Hence, the mobilisation of multiple TEs, including priority pollutants listed by the European Union (EU, 1976) and the United States Environmental Protection Agency (US Epa, 1998–1999), namely As, Cd, Cr, Cu, Ni, Sb, and Zn, through diverse traffic activities poses a significant threat to urban ecosystems.

Several factors, such as inadequate public transport systems, non-existent environmentally sustainable transport infrastructure, heavy reliance on private vehicles, roadside parking, numerous informal transport stops, slow-moving traffic, congested road junctions, and the absence of sidewalks and dedicated cycling lanes, often contribute to traffic congestion in cities worldwide, particularly in rapidly urbanising developing countries (Ackaah, 2019; Ayaz et al., 2023; Okosun et al., 2023; Rajé et al., 2018). These traffic patterns induce transient engine modes caused by frequent deceleration, stopping, prolonged queuing, and acceleration, which increase fuel consumption compared with steady-state driving (Höglund, 1994; Matzoros & Van Vliet, 1992; Thorpe & Harrison, 2008) and intensify both exhaust and non-exhaust emissions (Fussell et al., 2022; Limo et al., 2018). Such transient engine modes enhance the release of distinct elemental assemblages from both automobile and road components, affecting roadside soil TE profiles at a local scale (Fussell et al., 2022; Reeves et al., 2018; Sager, 2020). Therefore, while roadside soil contamination along highways is primarily driven by traffic volume, within cities, it is influenced by several other traffic-induced factors (Wang & Zhang, 2018). Consequently, discernible differences in soil pollution levels have been documented across urban functional zones (Suleymanov et al., 2025).

Numerous studies have examined the effects of various traffic-related factors on environmental pollution, focusing primarily on individual traffic variables and on larger metropolitan and capital cities or major economic centres (Hamzeh et al., 2011; Höglund, 1994; Lee et al., 2021; Li et al., 2022; Limo et al., 2018; Olowoyo et al., 2022; Pan et al., 2017; Wawer et al., 2015; Yang et al., 2015). On the contrary, relatively smaller cities that have experienced population growth and largely unplanned expansion have received little attention (Adimalla, 2020; Rajé et al., 2018; Wawer et al., 2015). In addition, the pollution footprint associated with multiple co-existing traffic conditions, for example, proximity to roads, road junctions, and parking areas, as well as the presence of highways and roadside transport stops, remains poorly understood. Addressing this knowledge gap is particularly relevant to non-metropolitan cities, where these conditions are prevalent and may collectively govern roadside soil pollution patterns.

Potchefstroom, classified as a secondary South African city (smaller than metropolises but larger than small towns; Donaldson et al., 2020; Marais & Cloete, 2017), was selected as the model urban system for this investigation. The specific objectives of this study were: (1) to compare the total concentrations of selected TEs in roadside soils with national and international soil quality benchmarks and to evaluate pollution levels in order to identify contaminating elements; (2) to identify the potential sources of TEs, thereby isolating the traffic conditions that contribute significantly to roadside soil contamination; and (3) to conduct ecological and human health risk assessments to examine the overall threat posed by polluted roadside soils, and determine how pollution and risk levels vary along city traffic gradients. This study hypothesises that commonly occurring traffic conditions deteriorate roadside soil quality and that the confluence of multiple traffic factors amplifies these effects in relatively smaller cities. This novel assessment approach, integrating multiple traffic factors, provides a preliminary framework for future studies in comparable urban settings to identify priority soil-polluting TEs, localities needing attention, and traffic conditions that may require management or modification to support urban sustainability.

## Materials and methods

### Study locality and site selection

**Fig. 1:**
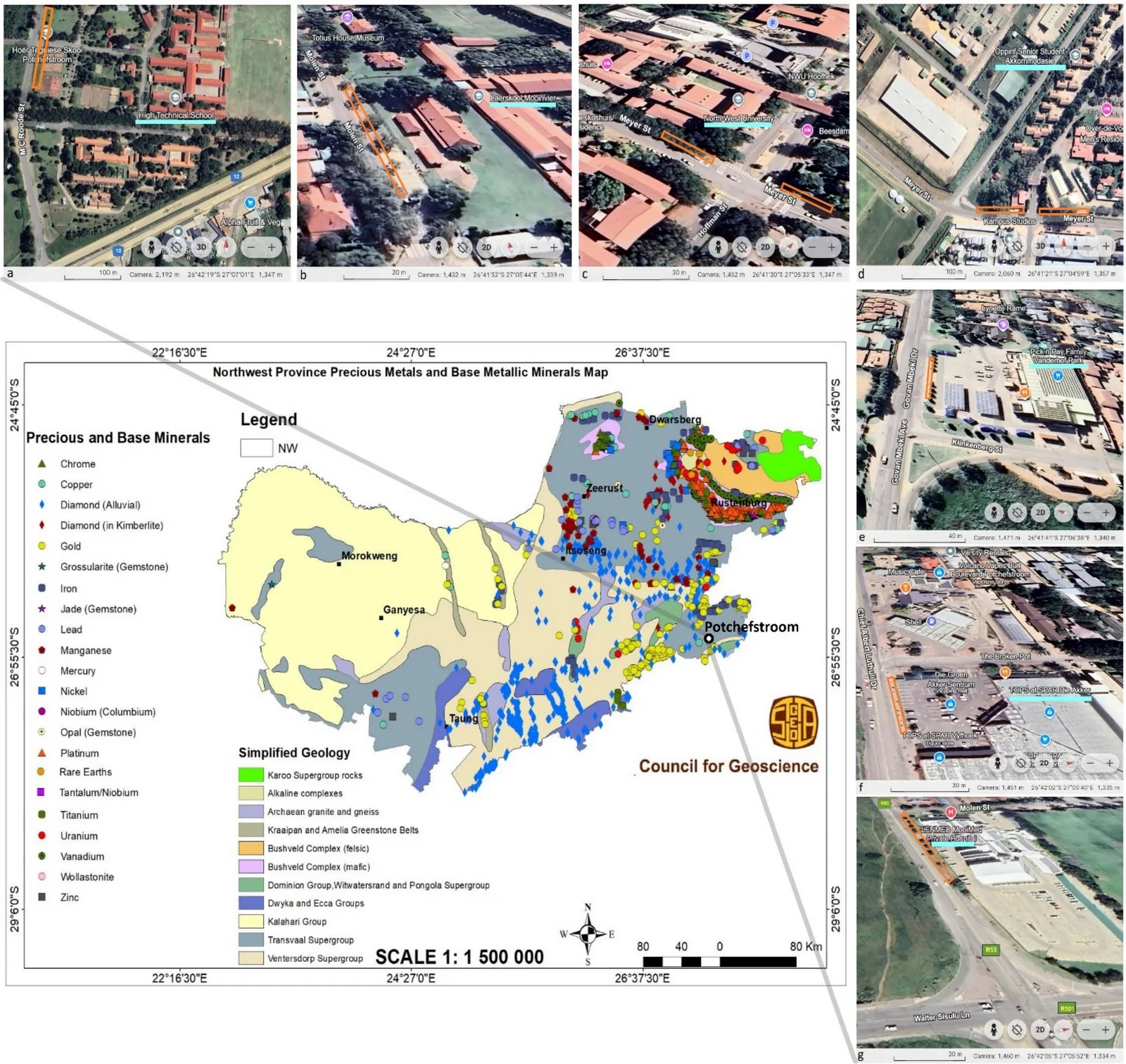
The mineral map of North West Province with the location of Potchefstroom, and the seven sampled sites in the city, (**a**) TS, (**b**) LS, (**c**) NG1, (**d**) NG2, (**e**) PN, (**f**) SP and (**g**) MH. Orange rectangles indicate sampling points. Source of mineral map of the North West Province: Council for Geoscience, South Africa (https://www.dmre.gov.za/Portals/0/Energy_Website/files/Mining/MiningInvestmentConference/Mining-and-Energy-Investment-Conference-Council-for-Geoscience.pdf)

The study area, Potchefstroom (26° 42′ 53″ S; 27° 05′ 49″ E), is situated in the North West Province of South Africa. The city experiences a cool, dry steppe climate with summer precipitation, receiving on average over 600 mm of rainfall annually. Summer temperatures generally exceed 32 °C, while subzero temperatures are common in winter (Cilliers & Bredenkamp, 1998). The province hosts a wide variety of economically valuable minerals (Fig. 1, mineral map inset), contributing nearly one-third to the provincial economy (Council for Geoscience, South Africa, CGS, 2022). However, Potchefstroom itself is not classified as a mining region (Annual Report, 2023/2024). The local economy mainly thrives on the manufacturing and services sectors, and the city is renowned as a university town (Annual Report, 2023/2024). The municipal population is largely composed of individuals aged 15 to 64 years (67%) and under 15 years (28%) (Annual Report, 2023/2024); hence, both these age groups were considered in this study.

The majority of households are formal (63%) (Annual Report, 2023/2024), therefore, various urban facilities located within formal settlement areas in the city and intersected by road networks were selected, including academic institutions [High Technical School (TS), Laerskool Mooirivier (LS), and North-West University (two gates: NG1 and NG2)], commercial centres [Pick n Pay (PN) and Superspar (SP)], and a medical facility [Lenmed MooiMed Private Hospital (MH)] (Fig. 1a–g). Entrances of these facilities serving as access points for pedestrians and public and private transport were targeted, as they constitute primary exposure zones of traffic-related TEs.

The site TS was located within 500 m of the national highway and intersected by a street leading into the city, and LS was traversed by a single street in a residential area. Site NG1, a primary university access point, was located near a busy traffic junction, whereas NG2, an auxiliary gate, was close to a relatively less congested junction. While PN was bounded on three sides by streets, SP and MH were adjacent to a busy road. Roadside areas outside TS and LS have been used for temporary car parking, and those of TS, LS, NG1, and NG2 function as informal transport stops. PN, MH, and SP had designated parking areas within the facilities, which were not directly on roadside soils. The parking area of MH was inside a fence. Roadside pavements were observed at LS, NG1, NG2, PN, and SP. Therefore, the seven sites were exposed to diverse traffic gradients due to differences in proximity to a highway (applicable for only TS), local roads (< 3 m), road junctions (30–500 m), and car parking areas (< 1 to 5 m), as well as the presence of a roadside transport stop. During sampling, vegetation was observed near the entrances of all sites, with cultivated hedgerows at SP. Trees and buildings were located within 50–150 m of all seven sites. At the time of sampling, cultivated ornamental plants were observed in roadside soils at NG2, with soil amendment application noted at SP.

### Sampling, preparation and analysis of roadside soil

A total of forty-six roadside soil samples were collected in 2024 from seven urban locations in Potchefstroom. Six sampling points, spaced 1 m apart, were established close to the entrances of each facility. At each point, five sub-samples were collected from a depth of 0.1 m (surface horizon) and within a 0.5 m radius, which were then combined to form a composite sample. All soil samples were collected within 3 m of the road edge (Sager, 2020; Werkenthin et al., 2014). This limit was chosen because (i) soils in this zone (within 5 m) typically contain the highest concentrations of technogenic materials, (ii) it is heavily impacted by traffic movement, (iii) deposition from runoff and splash water is greatest, and (iv) it represents areas where travellers wait for transport and where transport stops. Sampling points at the seven sites fell under three distance zones: < 1 m (TS), 1‒2 m (NG1, NG2, LS), and 2‒3 m (PN, SP, MH). Soil was collected using a stainless steel spade, which was cleaned with alcohol before sampling at each site. After air-drying thoroughly, soil samples were passed through a 2 mm sieve to remove coarser particles, plant materials and other debris, and were subsequently homogenised using a porcelain pestle and mortar (Hjortenkrans et al., 2006; Batwa‑Ismail et al., 2021; Nde et al., 2021). The powdered samples were then stored in numbered plastic containers at room temperature.

Roadside soil samples were delivered to the Eco-Analytica Laboratory in Potchefstroom, South Africa, for microwave assisted acid digestion (Epa 3051A method). To 200 mg (dry wt) of ground soil sample in individual Teflon tubes, 9 ml of 65% HNO₃ and 3 ml of 32% HCl in a 3:1 (v/v) ratio were added. The tubes were sealed and subjected to digestion in a high-performance system (Milestone Ethos UP, Maxi 44) for 20 min until the temperature reached 200 °C and was subsequently maintained for 15 min. After cooling, the volume in each tube was adjusted to 50 ml and total concentrations of As, Ba, Cd, Co, Cr, Cu, Mn, Ni, Pb, Sb, and Zn were quantified with an Inductively Coupled Plasma Mass Spectrometry (ICP-MS, Agilent 7500 series). The ICP-MS was equipped with CRC technology to minimise interferences. Soil samples were introduced through a micromist-type nebuliser, which reduces matrix effects and ensures a more robust plasma, and a standard quartz spray chamber. A solution containing 1 ppb of Li, Y, Ce, and Tl was used to optimise the instrument and achieve oxide and interference levels below 1.5% and maintain high sensitivity across the mass range (Adhikari & Struwig, 2024a).

Quality assurance and quality control in the laboratory setting were implemented by adhering to quality control schemes from Wageningen and Agrilasa, followed by Eco-Analytica Laboratory. Analytical grade Merck reagents, solvents and ultrapure deionised water from a Milli-Q analytical reagent grade water purification system were used for all analytical procedures. A multi-element standard calibration solution (mg/l) (ULTRASPEC, De Bruyn Spectroscopic Solutions, South Africa) containing the elements of interest was used to calibrate the instrument according to the Atomic Spectrometric Standard for the total quantitative method. For quality control, both blanks and standard checks were conducted every four sample intervals. Standard Multielement Certified Reference Materials (CRM, ULTRASPEC, De Bruyn Spectroscopic Solutions, South Africa) were analysed to ensure analytical accuracy. Duplicate samples were included at a defined frequency to assess analytical precision, and relative % differences were evaluated against laboratory control limits. Data accuracy was confirmed by obtaining a standard deviation of less than 5% between the values of the replicate and the original samples. All measurements were carried out in triplicate to ensure reproducibility (Nde et al., 2021).

### Pollution assessment

The Index of geo-accumulation (I_geo_) and contamination factor (CF) were estimated for each of the eleven TEs using Eq. (1) (Müller, 1969) and Eq. (2) (Håkanson, 1980), respectively. In both equations, C_n_ denotes the concentration (mg/kg) of element ‘n’ in the sampled roadside soil, whereas B_n_ represents the average concentration of the same element in the upper continental crust in Eq. (1) (Kabata-Pendias, 2011; Rudnick & Gao, 2014), and the global average soil concentration in Eq. (2) (Kabata-Pendias, 2011) (Table 1). The constant 1.5 in Eq. (1) accounts for lithological heterogeneity. Roadside soil pollution levels were classified according to Müller’s (1969) seven I_geo_ classes and Håkanson’s (1980) four-level CF classification scheme (Supplementary Information, Table S1).

1 $$I_{\mathrm{geo}}=log_{2} \left(\frac{C_n}{1.5\times B_n}\right)$$

2 $$CF=\frac{C_n}{B_n}$$

Pollution load index (PLI) was calculated as the geometric mean of the estimated CF for all eleven TEs (Eq. 3) (Tomlinson et al., 1980), reflecting roadside soil contamination from combined elements. The PLI values were interpreted according to Jiang et al. (2021) (Table S1).

3 $$PLI = \sqrt[11]{CF1\times CF2\times CF3\times \dots ..CF11}$$

### Risk assessment

#### Ecological risk

The potential ecological risk (Er) was calculated using Eq. (4), where CF^i^ and Tr^i^ represent the contamination factor (Eq. 2) and toxic response factor for element ‘i’. The Er was calculated for As, Cd, Co, Cr, Cu, Mn, Ni, Pb, and Zn, based on available Tr values of 10, 30, 5, 2, 5, 1, 5, 5, and 1, respectively (Håkanson, 1980; Li et al., 2022). The combined risk from the nine TEs was represented by the potential ecological risk index (RI) (Håkanson, 1980) (Eq. 4). Both Er and RI values were compared with recommended levels by Håkanson (1980) (Table S1).

4 $$RI= \sum\nolimits_{i=1}^{9}Er^i=\sum\nolimits_{i=1}^{9}Tr^i\times CF^i$$

#### Human health risk

This study followed US Epa (2002) models, as adjusted by the Framework for Management of Contaminated Land by the Department of Environmental Affairs, South Africa (DEA, 2010), to estimate non-carcinogenic risk and cancer risks for adults and children. Non-carcinogenic risks associated with ingestion and dermal exposure to roadside soil TEs were assessed using the hazard quotient (HQ) and the hazard index (HI) from individual (Eqs. 5‒7) and all elements (Eq. 8), respectively.

5 $$ADD_{\mathrm{ing}}=\frac{C\times IngR\times EF\times ED}{BW\times AT}\times CF$$

6 $$ADD_{\mathrm{der}}=\frac{C\times SA\times AF\times ABS\times EF\times ED}{BW\times AT}\times CF$$

7 $$HQ=\frac{ADD_{ing/der}}{RfD_{O/ABS}}$$

8 $$HI={\sum}_{i=1}^{11}HQ$$

In Eqs. (5 and 6), ADD_ing_ and ADD_der_ (mg/kg-day) present the average daily potential dose through direct ingestion and dermal contact, respectively. ‘C’ stands for TE concentration (mg/kg) in the roadside soil. RfD_O_ and RfD_ABS_ represent the reference doses for the evaluated TEs via ingestion and dermal exposure, respectively. The values of parameters are summarized in Table S2, while Table S3 presents RfD values. Both HQ and HI values exceeding 1 were interpreted to be hazardous, whereas values below this threshold were considered non-hazardous.

Cancer risk through oral exposure was assessed for As, Cd, and Pb, whereas for dermal contact, only As and Cd were considered (International Agency for Research on Cancer, IARC, 2021; Miletić et al., 2023). The potential lifetime cancer risk (CR) from each TE through individual exposure routes (Eqs. 9 and 10), as well as the total cancer risk (TCR), representing the combined risk from both pathways for element ‘i’ (Eq. 11), were determined.

9 $$CR_{ing}=ADD_{ing}\times SF_O$$

10 $$CR_{der}=ADD_{der}\times SF_{ABS}$$

11 $$TCR_i=CR_{ing,i}+CR_{der,i}$$

In these equations, CR_ing_ and CR_der_ represent cancer risks associated with ingestion and dermal exposure, respectively. The cancer slope factors (SF) used for ingestion (SF_O_) and dermal (SF_ABS_) exposure pathways are provided in Table S3. Cancer risk and total cancer risk values were evaluated based on the current toxicity assumption guidelines of the US Epa (2020), stating risks below 1 × 10⁻^6^ as negligible, those between 1 × 10⁻^6^ and 1 × 10⁻^4^ as acceptable, and risks exceeding 1 × 10⁻^4^ as unacceptable.

### Data analysis

Roadside soil element concentration data showed non-normal distribution (Shapiro–Wilk test, *p* < 0.05). Kruskal–Wallis tests with pairwise comparisons and Bonferroni corrections (for multiple groups), or Mann–Whitney U tests (for two groups) were performed to evaluate differences in means of (1) TE concentrations, (2) pollution indices, and (3) ecological risks among the sites, as well as (4) health risks across sites and between the two age groups (children and adults). The similarities among sites with regard to TE concentrations were investigated with hierarchical cluster analysis, using Ward’s linkage method and squared Euclidean distance. To explore the relationship between road proximity and roadside soil TE concentrations, linear regression analysis was performed considering the three distance groups: < 1 m (TS), 1‒2 m (NG1, NG2, LS), and 2‒3 m (PN, SP, MH). TE source identification incorporated principal component analysis (PCA) and Spearman’s rank correlation analysis. Varimax rotation and Kaiser normalisation were utilised for PCA. Furthermore, to explore the associations among TEs and traffic variables, sites were categorized based on considered traffic variables for PCA: presence (value 1) or absence (value 0) of (i) national highway (NH) [TS: NH = 1; other sites: NH = 0] and (ii) roadside transport stop (TT) [TS, LS, NG1, NG2: TT = 1; other sites: TT = 0]; proximity to (iii) car parking (CP) [TS, LS: CP = 4, roadside parking; SP: CP = 3, < 1 m; MH: CP = 2, < 2 m; PN: CP = 1, < 5 m; NG1, NG2: CP = 0, no nearby parking], (iv) road junction (RJ) [NG1, NG2: RJ = 5, < 30 m; PN, SP: RJ = 4, < 45 m; LS: RJ = 3, < 70 m; MH: RJ = 2, < 200 m; TS: RJ = 1, < 500 m], and (v) the nearest road (road proximity, RP) [TS: RP = 3, < 1 m; LS, NG1, NG2: RP = 2, 1‒2 m; PN, SP, MH: RP = 1, 2‒3 m]. Statistical significance was set at *p* < 0.05. Data analyses were performed using SPSS (IBM, v. 30.0, Armonk, New York, USA) and Microsoft Excel (Microsoft 365, Microsoft Corporation, Redmond, Washington, USA).

## Results and discussion

### Roadside soil trace element levels relative to soil quality guidelines

The mean element concentration ranges (mg/kg) followed the descending order: Mn (311.77–1283.69) > Ba (55.93–185.32) > Cr (73.35–182.29) > Zn (50.30–199.53) > Ni (30.20–54.30) > Pb (16.60–45.84), > Cu (28.99–42.16) > Co (8.44–19.58) > As (3.76–9.07) > Sb (0.64–1.20) > Cd (0.07–0.16) across the sites (Table 1). In general, mean concentrations remain lower than the following international quality guidelines for agricultural soils, Maximum Allowable Concentration (MAC) and Trigger Action Value (TAV) (Table 1) (Kabata-Pendias, 2011). For Mn, neither of these standards was available. However, mean Mn concentrations at most sites fell within the range recommended for uncontaminated soils (400–900 mg/kg) (Pavilonis et al., 2015), except at TS, where the mean (946 mg/kg) was marginally higher than the upper limit of this range. Furthermore, mean concentrations of Cd, Cr, Cu, Ni, Pb, and Zn at the sampled sites remained below the Total Maximum Thresholds (TMT) recommended for surface soils in South Africa (Table 1) (Herselman et al., 2005). However, further comparison of average soil TE levels with the South African Soil Screening Value 1 (SSV1), which is protective of risks to humans and the environment covering all land uses (DEA, 2013), revealed that mean Cu concentrations at all sites were nearly double or more than the respective threshold (Table 1). Similarly, mean Pb concentrations exceeded the SSV1 by up to twofold at most sites (86%), except SP, where a lower mean was recorded. Among the other TEs for which SSV1 was available, the mean Mn concentration at TS and that of As at TS and LS exceeded the respective thresholds (Table 1).

**Table 1:** Trace element concentrations (mg/kg) in sampled roadside soils and South African and international standards

Sampling sites		As	Ba	Cd	Co	Cr	Cu	Mn	Ni	Pb	Sb	Zn
TS	Mean	9.07	185.32	0.09	19.58	157.23	39.21	946.47	54.33	31.82	1.78	155.36
	Max	12.18	237.39	0.10	21.56	182.29	47.10	1162.00	90.50	37.30	2.54	207.83
	Min	6.54	138.41	0.07	15.91	136.60	28.80	781.52	40.32	26.60	0.90	70.26
	SD	2.26	37.93	0.01	2.04	15.85	5.95	160.15	18.42	4.16	0.53	48.25
LS	Mean	6.42	121.80	0.15	13.77	122.41	30.82	643.61	36.55	40.69	0.92	104.30
	Max	9.63	145.31	0.35	18.35	153.27	42.27	782.54	60.35	90.17	1.22	173.41
	Min	4.65	93.28	0.07	10.90	95.05	21.73	527.11	26.46	21.18	0.58	61.43
	SD	1.69	19.99	0.10	3.18	21.66	9.15	107.72	13.04	25.08	0.23	53.42
NG1	Mean	5.20	143.72	0.11	16.17	120.53	29.01	737.09	45.06	40.28	1.03	91.84
	Max	6.48	266.41	0.17	22.75	128.74	34.05	1283.69	71.44	67.55	1.99	113.97
	Min	3.57	89.89	0.06	12.29	112.96	22.68	521.06	35.98	18.36	0.50	58.74
	SD	1.09	64.92	0.04	3.56	6.05	3.65	282.63	13.19	20.70	0.52	20.83
NG2	Mean	4.78	105.41	0.10	11.63	107.36	33.59	568.45	30.22	37.88	0.92	110.36
	Max	6.48	128.27	0.13	12.82	130.23	39.50	697.25	38.31	50.33	1.32	195.71
	Min	3.09	86.07	0.06	10.89	89.39	26.46	446.70	21.11	25.86	0.62	58.43
	SD	1.25	15.24	0.03	0.92	16.60	4.39	82.42	6.18	9.63	0.32	57.38
PN	Mean	5.25	112.06	0.11	11.44	98.75	42.16	582.89	41.11	24.70	1.13	99.56
	Max	6.45	148.14	0.32	13.58	108.06	120.93	826.26	46.58	36.48	2.03	134.91
	Min	4.31	93.08	0.05	10.66	87.84	24.62	471.72	31.39	19.65	0.62	74.10
	SD	0.91	19.90	0.10	1.07	8.44	38.61	126.48	6.88	6.02	0.51	20.48
SP	Mean	3.76	55.93	0.16	8.44	87.15	28.99	321.00	33.31	16.65	0.64	114.65
	Max	5.51	68.01	0.46	14.69	99.04	42.00	377.61	53.72	37.80	1.16	199.53
	Min	2.27	48.64	0.05	5.38	73.35	21.68	283.36	23.95	7.32	0.41	50.30
	SD	1.29	6.63	0.16	3.40	11.01	7.34	30.85	11.51	11.04	0.28	60.00
MH	Mean	5.76	97.16	0.07	11.50	99.00	33.49	573.78	31.49	45.84	1.20	87.97
	Max	7.15	118.75	0.13	12.92	115.35	47.23	818.76	46.69	171.09	3.38	132.51
	Min	4.05	65.41	0.04	9.11	87.11	23.66	422.42	25.22	16.20	0.61	70.09
	SD	1.06	23.16	0.03	1.51	9.14	10.33	150.81	7.74	61.49	1.08	22.53
Earth’s upper crust average^a,b^		4.80	624	0.09	17.30	92	28	900	47	17	0.40	67
World soil average^b^		6.82	460	0.41	11.30	59.50	38.90	488	29	0.67	27	70
MAC^b^		15‒20	NA	1‒5	20‒50	50‒200	60‒150	NA	20‒60	20‒300	10	100‒300
TAV^b^		10‒65	400‒600	2‒20	30‒100	50‒450	60‒500	NA	75‒150	50‒300	10‒20	200‒1500
TMT^c^		NA	NA	3	NA	350	120	NA	150	100	NA	200
SSV1^d^		5.8	NA	7.5	300	NA	16	740	91	20	NA	240

Max, maximum; Min, minimum; SD, standard deviation (n = 6); MAC, Maximum Allowable Concentration; TAV, Trigger Action Value; TMT, Total Maximum Threshold (South Africa); SSV1, Soil Screening Value 1 (South Africa); NA not available. ^a^ Rudnick & Gao, 2014; ^b^ Kabata-Pendias, 2011; ^c^ Herselman et al., 2005; ^d^ DEA, 2013

Concentrations of selected TEs in roadside soils from the investigated localities exhibit considerable variation when compared with those reported for inner urban zones and highway corridors in cities of differing sizes worldwide. For example, relative to the present study, Werkenthin et al. (2014) reported higher Cd and Zn, lower Cr, and comparable Cu, Ni, and Pb concentrations in several European cities, while in Wuhan, China, Zhang et al. (2015) documented generally higher Cr, Cu, and Zn, but lower Cd and Pb concentrations. In Hangzhou, Wang and Zhang (2018) observed higher Cd, Mn, Pb, and Zn, lower Cr and Co, and comparable Cu levels. Along the highways, Poznanović Spahić et al. (2025) reported predominantly higher As, Co, and Cd and comparable Pb and Sb concentrations in Vojvodina, Serbia, whereas An et al. (2022) found higher As and Zn, lower Cr and Cu, and similar concentrations of Co and Pb in the Tibetan Plateau.

Variations in roadside soil elemental profiles among cities, irrespective of their size, reflect the critical importance of various locality-specific aspects. In this context, the role of climate, topography, vegetation, and land use should be addressed in future studies (An et al., 2022; Jiang et al., 2021; Reeves et al., 2018). Of particular importance is air movement, which governs the dispersion, deposition, and spatial distribution of pollutants in both upwind and downwind directions (Korzeniowska, 2022; Li et al., 2018; Matzoros & Van Vliet, 1992; Zhang et al., 2012). This is especially relevant for volatile metalloids, such as As and Sb, both of which can occur in gaseous and particulate forms, thereby facilitating wider dispersion and contamination ranges (Hjortenkrans et al., 2006; Luilo et al., 2014; Sparks, 2005).

In Potchefstroom, Cu and Pb at most sites, together with As and Mn at a few sites, drive potential progressive roadside soil degradation. This finding is consistent with studies from cities of varying sizes, for instance, in China (Wang & Zhang, 2018; Zhang et al., 2015), Greece (Christoforidis & Stamatis, 2009), Korea (Lee et al., 2021), Russia (Suleymanov et al., 2025), South Africa (Krüger et al., 2019), and Sweden (Hjortenkrans et al., 2006). These studies conclude that anthropogenic activities, including traffic, dominate the enrichment of these TEs across diverse environmental matrices, particularly roadside soils. For Potchefstroom, this finding suggests a potential risk of food chain contamination in roadside environments, consistent with similar findings reported for another relatively small town in the same province (Adhikari & Struwig, 2024b).

**Fig. 2:**
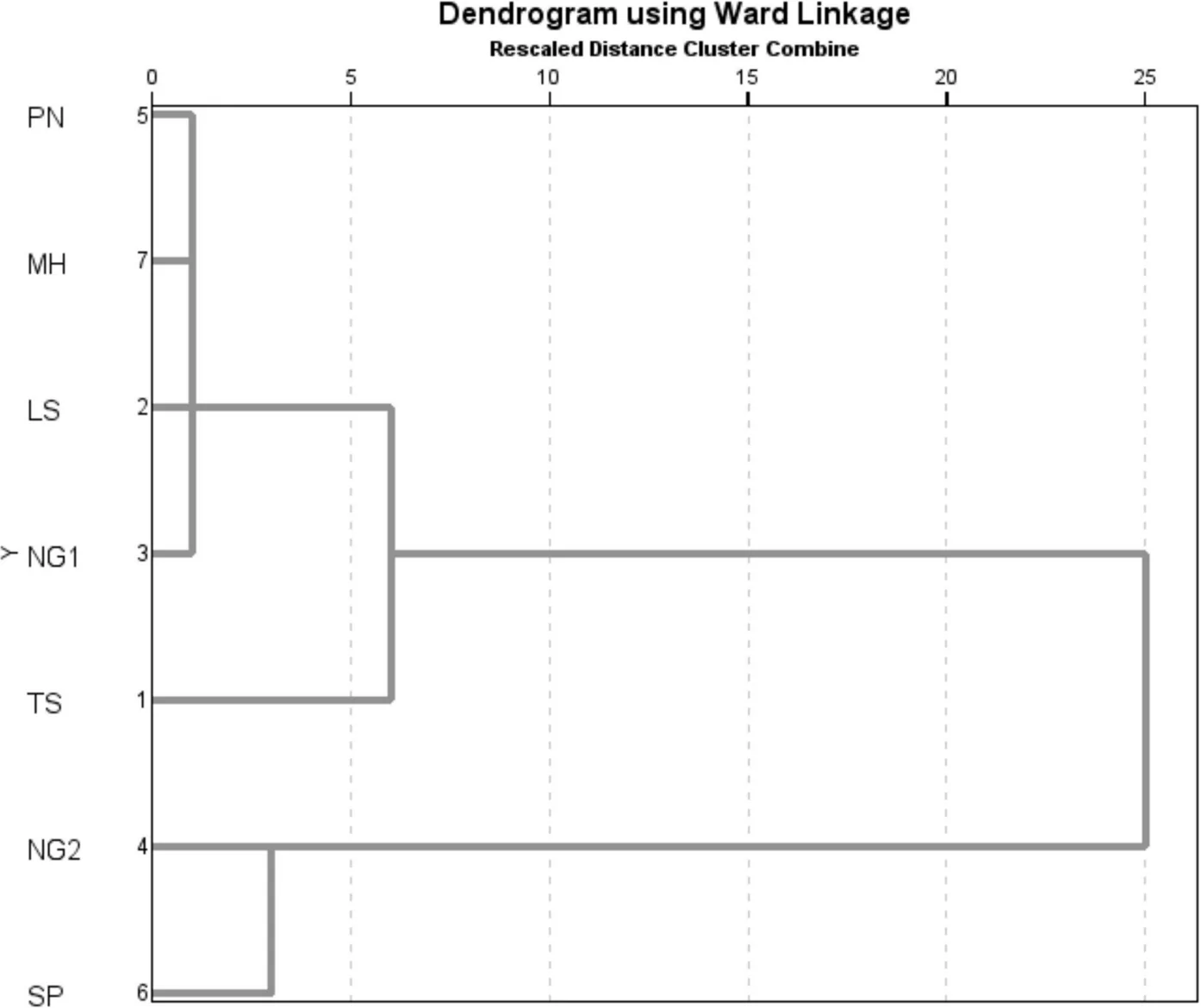
Hierarchical cluster analysis dendrogram of the seven sampling sites based on the concentrations of evaluated trace elements in roadside soil

The sites TS and SP exhibited the highest and lowest concentrations of multiple TEs, respectively. The highest concentrations of 36% of the TEs, namely As, Cr, Ni, and Zn, were recorded at TS, followed by NG1 (27%, Ba, Co, Mn) and MH (18%, Pb, Sb). In addition, SP (9%, Cd) and PN (9%, Cd) each showed the highest level of a single element (Table 1). Comparison of means revealed that TS had significantly higher (*p* < 0.05) levels of As, Ba, Co, Cr, Mn, and Sb than SP; As and Ni compared to NG2; and Co and Cr compared with PN and MH. Apart from TS, soils at NG1 had significantly higher mean Ba, Co, and Mn levels compared to SP, and LS had significantly higher mean Ba levels than SP. The hierarchical cluster analysis dendrogram (Fig. 2) further elucidated TE distribution patterns by isolating TS as a distinct cluster in the middle based on its relatively higher roadside soil elemental concentrations. Sites in the top cluster were characterised by generally moderate to high TE levels. The site TS displayed a closer association with this cluster. The bottom cluster included SP and NG2, both of which exhibited comparatively lower soil TE contents.

**Fig. 3:**
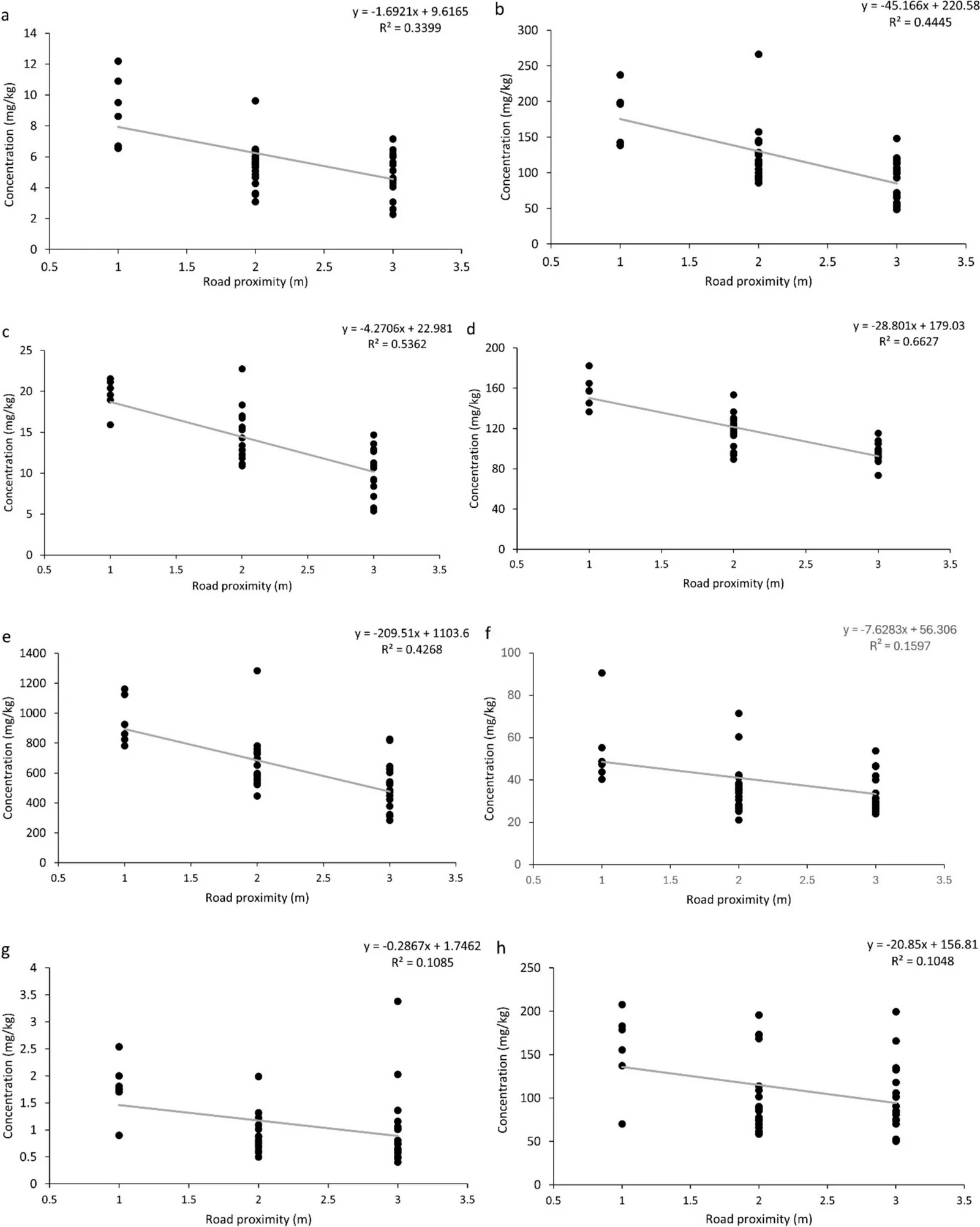
Significant relationships (*p* < 0.05) between road proximity and concentrations of the following trace elements in roadside soil: (**a**) arsenic, (**b**) barium, (**c**) cobalt, (**d**) chromium, (**e**) manganese, (**f**) nickel, (**g**) antimony and (**h**) zinc

Regression analyses revealed positive relationships between all assessed TEs and road proximity, with statistically significant (*p* < 0.05) increase for 73% of elements, that is, for all TEs except Cd, Cu, and Pb (Fig. 3). Stronger relationships (*p* < 0.001) were observed for As, Ba, Co, Cr, Mn, and Ni (Fig. 3a–f) compared with Sb and Zn (*p* < 0.05) (Fig. 3g, h).

The observed enhancing effect of road proximity on roadside soil TE levels in this study aligns with several international, city-oriented studies focused on multiple common elements, including As, Cr, Ni, Pb, Sb, and Zn (Carrero et al., 2013; Korzeniowska, 2022; Luilo et al., 2014; Negahban & Mokarram, 2021; Poznanović Spahić et al., 2025). This explains the generally higher concentrations of several TEs at TS (< 1 m) and NG1 and LS (1–2 m), all located closest to the road edge. This further relates well to comparatively high year-round traffic loads at these sites (Batwa-Ismail et al., 2021; Bukowiecki et al., 2009; Fussell et al., 2022; Thorpe & Harrison, 2008; Wang & Zhang, 2018).

Sites (MH, SP, and PN) located furthest from the road edge exhibited generally lower concentrations of most TEs, with the highest levels of a few elements. These elevated levels may be attributed to specific local influencing factors. Previous studies have suggested that soil type, infiltration rates, roadside topography influencing water flow and atmospheric deposition, and soil management practices contribute to such differential patterns (An et al., 2022; Negahban & Mokarram, 2021; Poznanović Spahić et al., 2025; Wang et al., 2021; Werkenthin et al., 2014). Particularly, SP, which was impacted by the same relatively busy road as MH, had notably lower TE levels among the seven sites. This could be partially attributed to compost application, which has been recommended as an affordable remediation strategy for polluted roadside soil due to its ability to enhance TE immobilisation through alterations in key soil properties, including mineral composition, organic matter content, microbial activity, and pH (US Epa, 1999; Weindorf et al., 2013). Furthermore, elevated Cd levels at SP could be linked to fertiliser addition (Liao et al., 2022; Sager, 2020). Conversely, the lower soil TE concentrations at NG2, located within 2 m of the road edge, may be associated with relatively low traffic density and the possible application of compost to cultivated plants.

In addition, the presence of pavement may have suppressed dust and reduced particle resuspension, thereby contributing to low soil element levels at NG2 and SP (Reeves et al., 2018). The presence of trees near TS, PN, LS, and NG1, as well as the proximity of NG1 and LS to buildings in different directions, may have partially modified surface-level airflow patterns, thereby manipulating particulate matter dispersion on a local scale (Baldauf, 2017; An et al., 2022; Wang et al., 2021). This observation also accords with the relatively low TE concentrations in soils at MH and SP, where open grounds opposite the adjacent road possibly facilitated dust dispersion. Moreover, hedgerows at SP likely play a critical role by reducing dust resuspension, trapping particulate matter and accumulating TEs, which should be investigated (Biocca et al., 2024). Evaluation of factors like soil properties and road age is also essential to further explicate roadside soil TE distribution patterns in the city (Carrero et al., 2013; Reeves et al., 2018; Werkenthin et al., 2014).

### Source identification of trace elements in roadside soil

The correlation matrix based PCA extracted four principal components, with the first component (PC1) accounting for a larger percentage of total data variance compared to the other PCs, indicating its greater influence on data variability (Table 2). Loading values greater than 0.45 were considered significant in this study. Eight out of the eleven TEs (73%, excluding Cu, Cd, and Pb), along with road proximity (RP), transport stop (TT), and national highway (NH), displayed high positive loadings on PC1. In addition to PC1, As, Zn, and national highway exhibited high positive loadings on PC2, alongside car parking (CP) and a negative loading of road junction (RJ). High positive loadings of Cu and Sb, with a high negative loading of Cd, were observed on PC3. On PC4, only Zn and Cd had moderate positive loadings, along with a high negative loading of Pb. Generally, negative or very low loadings of road junction (RJ) on PCs indicate a weak contribution to data variability. The substantially high positive loadings of several TEs and their associations with one or more traffic variables suggest contributions from multiple shared sources (Table 2).

**Table 2:** Varimax rotated principal component analysis matrix of the evaluated variables (trace elements and traffic factors)

Variables	PC1	PC2	PC3	PC4
RP	0.961	0.170	−0.028	−0.020
Cr	0.927	0.334	0.161	−0.049
Co	0.924	0.184	0.245	−0.132
Ba	0.903	0.122	0.361	−0.117
TT	0.885	−0.127	−0.317	−0.213
Mn	0.872	0.168	0.391	−0.201
Ni	0.766	0.173	0.365	0.301
As	0.703	0.581	0.379	−0.101
NH	0.671	0.520	0.392	0.261
Zn	0.579	0.484	0.146	0.519
CP	0.116	0.941	−0.202	0.203
RJ	−0.179	−0.899	−0.377	0.123
Cu	0.107	0.100	0.847	0.284
Cd	−0.140	0.064	−0.792	0.481
Sb	0.586	0.394	0.702	−0.078
Pb	0.261	−0.022	0.018	−0.961
Eigenvalue	9.49	2.42	1.95	1.24
Variance (%)	59.34	15.18	12.18	7.80
Total variance (%)	59.34	74.52	86.70	94.50

CP, car parking; NH, national highway; RJ, road junction; RP, road proximity; TT, transport stop

Spearman’s rank correlation matrix revealed a high number of significant positive correlations, mainly at *p* < 0.01 (Table 3), for nine out of eleven TEs (82%), excluding only Cd and Zn. This further suggests common sources for most elements. Among the TEs, Sb and Pb exhibited the highest number of significant correlations. While Sb was significantly correlated with all elements except Cd, Pb was significantly correlated with all TEs except Ni and Zn. Of the remaining TEs, higher numbers of significant correlations were observed for As, Ba, Co, Cr, and Mn (seven each), followed by Ni (six) and Cu (four). On the other hand, Cd and Zn exhibited the fewest significant positive correlations. Cd was correlated with Ni and Pb, and Zn with Cu and Sb (Table 3).

**Table 3:** Spearman’s rank correlation matrix of trace elements in roadside soils

	As	Ba	Cd	Co	Cr	Cu	Mn	Ni	Pb	Sb
Ba	0.566^**^	–								
Cd	0.155	0.088	–							
Co	0.538^**^	0.767^**^	0.266	–						
Cr	0.585^**^	0.641^**^	0.262	0.711^**^	–					
Cu	0.474^**^	0.265	0.118	0.161	0.280	–				
Mn	0.683^**^	0.917^**^	0.091	0.784^**^	0.672^**^	0.297	–			
Ni	0.133	0.411^**^	0.338^*^	0.597^**^	0.405^**^	−0.016	0.352^*^	–		
Pb	0.400^**^	0.467^**^	0.449^**^	0.440^**^	0.506^**^	0.443^**^	0.490^**^	0.035	–	
Sb	0.587^**^	0.513^**^	0.183	0.412^**^	0.468^**^	0.476^**^	0.481^**^	0.310^*^	0.565^**^	–
Zn	0.272	0.084	0.052	0.090	0.215	0.661^**^	0.112	0.168	0.201	0.444^**^

Significance level: ^**^ *p* < 0.01; ^*^ *p* < 0.05

Global research has documented similar relationships among elements in urban traffic impacted soils, including Mn with Co, Cr, and Ni, as well as Co–Ni in various European and Asian countries (Wawer et al., 2015); Cr–Co–Ni and As–Cu–Pb on the Tibetan Plateau (An et al., 2022); Cd–Pb in a Greek city (Christoforidis & Stamatis, 2009); As–Sb in a city in Spain (De Miguel et al., 2017); and Cu–Sb–Zn in multiple Swedish cities (Hjortenkrans et al., 2006). Nonetheless, consistent with this study, discernible differences in correlation patterns among cities have been frequently reported (An et al., 2022; De Miguel et al., 2017; Fussell et al., 2022; Werkenthin et al., 2014). As discussed in the previous sections, this finding further emphasises the importance of site-specific factors, including prevailing traffic patterns in the investigated areas of the city.

Associations among most of the evaluated TEs with one or more examined traffic variables confirm vehicular activities as a common and predominant anthropogenic source. Identification of road proximity as a key influencing factor is in agreement with the regression outcomes discussed earlier. The presence of a roadside transport stop is indicated as another high-impact factor, which may primarily be related to emissions from transient driving modes at these locations, alongside automobile fuel combustion generated TEs, such as As (Lee et al., 2021). In particular, the release of Cr, Ni, Sb, and Zn may be linked to vehicle deceleration and stop related brake wear; Co, Cr, Mn, and Ni could have also originated from tire wear, whereas Cr, Ni, and Zn may derive from the gradual degradation of pavement materials (An et al., 2022; Carrero et al., 2013; Fussell et al., 2022; Hjortenkrans et al., 2006; Kadi, 2009; Yang et al., 2015).

Consistent with the findings of this study, past investigations have reported soil contamination by several common elements along highways, including As, Cr, Ni, Sb, and Zn (An et al., 2022; Batwa-Ismail et al., 2021; Cal-Prieto et al., 2001; Kadi, 2009; Poznanović Spahić et al., 2025). In this study, this finding is driven solely by the potential impact of the national highway on soil TE contents at TS, which requires future attention. The positive associations of only As and Zn with car parking suggest that this variable probably acts as an auxiliary source. In this case, tire wear, given the use of Zn in tire vulcanisation and the likely presence of both TEs in fuel exhaust, may have enhanced their accumulation in soils in or around parking areas (Fussell et al., 2022; Hjortenkrans et al., 2006; Lee et al., 2021; Limo et al., 2018; Yang et al., 2015).

In the present study, the potential influence of proximity to road junctions may have been obscured by more dominant traffic factors at individual sites. For example, the presence of all three high-impact traffic conditions at TS could have overridden any effects associated with this site being located furthest from a road junction. Future research may provide more definitive insights into the relative influence of this factor on soil contamination. Globally, studies assessing the pollution footprints of these traffic-related variables are sufficient, with the exception of transport stops, for which no comparable evidence is available, highlighting a knowledge gap that should be addressed by future research (An et al., 2022; Batwa-Ismail et al., 2021; Cal-Prieto et al., 2001; Hjortenkrans et al., 2006; Limo et al., 2018).

Taken together, the strong associations among road proximity, transport stops, national highway, and 73% of the assessed TEs emphasise the predominance of multi-point traffic-related sources, complicating the attribution of precise TE origins at these sites, as also reported in past studies (Hjortenkrans et al., 2006; Thorpe & Harrison, 2008). This further explains the generally elevated roadside soil TE concentrations at certain sites, particularly TS, NG1, and LS, all of which are influenced by multiple traffic conditions, and highlights the combined effect of traffic variables on roadside soil TE profiles, thereby supporting the hypothesis. These results suggest that non-exhaust emissions associated with transient vehicular movements may contribute substantially to TE accumulation in roadside soils in relatively small cities (Matzoros & Van Vliet, 1992; Thorpe & Harrison, 2008). In addition, these findings highlight the need to incorporate these often underestimated traffic-associated factors into roadside soil pollution assessments in areas with similar traffic patterns.

That said, at all sites, automobile exhaust fumes linked to fossil fuels, engine oils, and catalysts are also presumably intensified by transient vehicular movement. For example, fuel exhaust containing Pb and traces of As, Cd, Cr, Cu, and Ni, fuel additives such as methylcyclopentadienyl manganese tricarbonyl (MMT), and the presence of Zn as an antioxidant in engine oil (Hjortenkrans et al., 2006; Krüger et al., 2019; Lee et al., 2021). Furthermore, certain TEs are typically associated with specific particle size fractions emitted from different fuel types, such as diesel derived nanoparticles containing Ba, Cd, Pb, Sb, and Zn, whereas ultrafine particles emitted by gasoline are enriched with Cu and Mn (Kuklová et al., 2022). Other potential contributions include Cr from the wear of steel vehicle parts and Cu from transient vehicle movement (Fussell et al., 2022). Associations between Cu–Sb and Zn–Cd further indicate natural origins, as well as the influence of applied agrochemicals and amendments (Laker, 2023; Liao et al., 2022; Sager, 2020).

In the present study, a set of TEs, namely Pb, Cd, Cu, and Zn, which are widely linked to both traffic-related and natural sources (Grzebisz et al., 2002; Kadi, 2009; Wawer et al., 2015; Werkenthin et al., 2014), exhibited inconsistency between correlation and PCA outcomes, likely due to multiple impacting factors. For Pb, this may be attributed to factors such as its presence in varying proportions in different fossil fuels, a comparatively minor contribution from brake wear, and legacy accumulation patterns (Batwa-Ismail et al., 2021; Carrero et al., 2013; Thorpe & Harrison, 2008). Additionally, its propensity to form stable complexes with soil organic matter, together with its relatively rapid transport into the soil pore system, may have further influenced its distribution patterns (Werkenthin et al., 2014). Similarly, Cd, recognised as one of the most mobile TEs, may accumulate in surface soils but can also be transported rapidly to subsurface horizons depending on prevailing edaphic conditions (Sager, 2020). Moreover, all four TEs are typically present in labile forms in soil, with more than 40% potentially occurring in mobile fractions depending on soil properties and land use (Ajmone-Marsan & Biasioli, 2010; Christoforidis & Stamatis, 2009; Liao et al., 2022; Werkenthin et al., 2014). Collectively, these factors may have influenced the distribution patterns of these TEs in roadside soils across both spatial and temporal scales, thereby affecting detectable relationships with other TEs and traffic variables.

### Pollution assessment

The mean I_geo_ indicated Pb and Sb as the prevalent contaminating elements in roadside soils (Table 4, Table S5). In general, for both TEs, an uncontaminated to moderately contaminated status (class 1) was documented at most sites, except TS, which showed class 2 contamination for Sb, and at PN and SP, which showed class 0 (I_geo_ < 0, uncontaminated) status for Pb. For As, Cr, and Zn, class 1 contamination was observed only at TS, while other sites showed uncontaminated status (Table 4). Whereas for the rest of the TEs (55%, Ba, Cd, Co, Cu, Mn, and Ni), an uncontaminated status was indicated (Table 4, Table S5). Altogether, nearly half of the assessed TEs (five out of eleven, 45%) showed some degree of contamination across sites. Based on the highest values, TEs followed the descending order, with maximum I_geo_ value and corresponding site in brackets: Pb (2.75, MH) > Sb (2.50, MH) > Cd (1.78, SP) > Cu (1.53, PN) > Zn (1.05, TS) > As (0.76, TS) > Cr (0.40, TS) > Ni (0.36, TS) > Mn (0.07, NG1) > Co (‒0.27, SP) > Ba (‒1.98, TS) (Table 4). Kruskal–Wallis tests revealed significant differences (*p* < 0.05) in mean I_geo_ among sites, with TS exhibiting significantly higher values for As compared to SP and NG2, for Ba and Sb relative to SP, for Co and Cr compared to SP, PN, and MH, and for Ni than NG2. Apart from TS, significantly higher mean I_geo_ was observed at NG1 for Ba, Co, and Mn relative to SP, and at LS for Ba compared to SP.

**Table 4:** Index of geo-accumulation (I_geo_) values for roadside soil trace elements

Sampling sites		Index of geo-accumulation (I_geo_)										
		As	Ba	Cd	Co	Cr	Cu	Mn	Ni	Pb	Sb	Zn
TS	Mean	0.30	−2.36	−1.35	−0.41	0.18	−1.20	−1.06	−0.43	0.31	1.51	0.49
	Max	0.76	−1.98	−0.41	−0.27	0.40	0.17	−0.22	0.36	0.55	2.08	1.05
	Min	−0.14	−2.76	−0.86	−0.71	−0.01	−0.54	−0.79	−0.81	0.06	0.58	−0.52
	SD	0.37	0.31	0.16	0.16	0.14	0.23	0.24	0.42	0.19	0.50	0.51
LS	Mean	−0.20	−2.96	−0.79	−0.94	−0.19	−1.59	−1.61	−1.01	0.50	0.58	−0.15
	Max	0.42	−2.69	1.38	−0.50	0.15	0.01	−0.79	−0.22	1.82	1.03	0.79
	Min	−0.63	−3.33	−0.94	−1.25	−0.54	−0.95	−1.36	−1.41	−0.27	−0.04	−0.71
	SD	0.35	0.24	0.85	0.32	0.26	0.42	0.24	0.46	0.72	0.39	0.68
NG1	Mean	−0.50	−2.81	−1.13	−0.71	−0.20	−1.63	−1.47	−0.69	0.50	0.64	−0.23
	Max	−0.15	−1.81	0.32	−0.19	−0.10	−0.30	−0.07	0.02	1.41	1.73	0.18
	Min	−1.01	−3.38	−1.17	−1.08	−0.29	−0.89	−1.37	−0.97	−0.47	−0.27	−0.77
	SD	0.32	0.57	0.52	0.30	0.07	0.19	0.47	0.36	0.75	0.68	0.36
NG2	Mean	−0.63	−3.16	−1.26	−1.16	−0.38	−1.42	−1.79	−1.25	0.53	0.55	−0.08
	Max	−0.15	−2.87	−0.05	−1.02	−0.08	−0.09	−0.95	−0.88	0.98	1.14	0.18
	Min	−1.22	−3.44	−1.14	−1.25	−0.63	−0.67	−1.60	−1.74	0.02	0.04	−0.77
	SD	0.40	0.21	0.46	0.11	0.22	0.20	0.21	0.31	0.37	0.49	0.71
PN	Mean	−0.47	−3.08	−1.38	−1.19	−0.49	−1.39	−1.77	−0.80	−0.08	0.81	−0.10
	Max	−0.16	−2.66	1.22	−0.93	−0.35	1.53	−0.71	−0.60	0.52	1.76	0.42
	Min	−0.74	−3.33	−1.35	−1.28	−0.65	−0.77	−1.52	−1.17	−0.38	0.04	−0.44
	SD	0.25	0.24	1.00	0.13	0.12	0.90	0.28	0.26	0.31	0.62	0.29
SP	Mean	−1.01	−4.07	−1.07	−1.71	−0.67	−1.66	−2.61	−1.14	−0.83	0.01	−0.06
	Max	−0.39	−3.78	1.78	−0.82	−0.48	0.00	−1.84	−0.39	0.57	0.95	0.99
	Min	−1.67	−4.27	−1.45	−2.27	−0.91	−0.95	−2.25	−1.56	−1.80	−0.56	−1.00
	SD	0.51	0.16	1.29	0.53	0.19	0.35	0.13	0.45	0.83	0.54	0.83
MH	Mean	−0.34	−3.31	−1.77	−1.19	−0.48	−1.47	−1.80	−1.19	0.19	0.69	−0.29
	Max	−0.01	−2.98	−0.06	−1.01	−0.26	0.17	−0.72	−0.59	2.75	2.50	0.40
	Min	−0.83	−3.84	−1.59	−1.51	−0.66	−0.83	−1.68	−1.48	−0.65	0.01	−0.52
	SD	0.28	0.37	0.55	0.20	0.13	0.45	0.37	0.31	1.28	0.92	0.32

Max, maximum; Min, minimum; SD, standard deviation (n = 6)

**Fig. 4:**
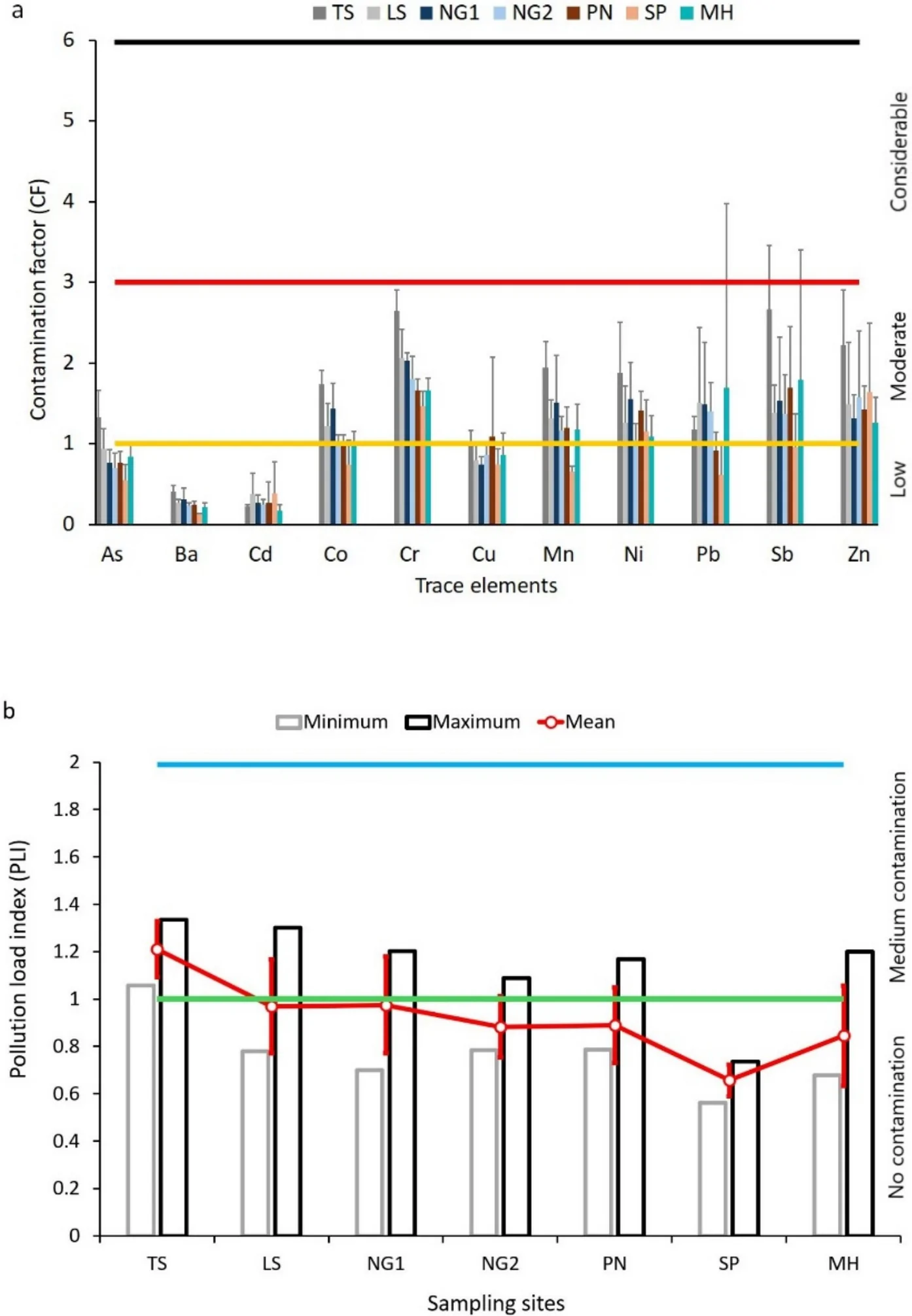
**a** Contamination factor (CF) for roadside soil trace elements, and (**b**) pollution load index (PLI) for the seven urban sites. Error bars represent standard deviation (n = 6). Thresholds: CF ‒ yellow line, low contamination; red line, moderate contamination; black line, considerable contamination; PLI ‒ green line, no contamination; blue line, moderate contamination

With regard to CF, the averages for Cr, Ni, and Zn fell within the moderate contamination range (1 < CF < 3) at all sites (Fig. 4a, Table S6). In addition, the mean CF for Co, Mn, and Sb indicated moderate contamination at 86% of sites, excluding only SP, which showed low contamination (CF < 1). For Pb, CF was above 1 at most sites (71%), except SP and PN, both of which had lower values. The mean CF for As at TS and for Cu at TS and PN exhibited moderate contamination, while the remaining sites showed low contamination. For Ba and Cd, mean CF remained low at all seven sites (Fig. 4a, Table S6). Altogether, 64% of the assessed TEs (seven out of eleven) showed substantial contamination at up to 86% of sites (six out of seven). According to the highest CF values, elements were ranked as follows, with CF value and corresponding site in brackets: Pb (6.34, MH) > Sb (5.05, MH) > Ni (3.12, TS) > Cu (3.11, PN) > Cr (3.06, TS) > Zn (2.97, TS) > Mn (2.63, NG1) > Co (2.01, NG1) > As (1.78, TS) > Cd (1.13, SP) > Ba (0.58, NG1) (Fig. 4a). Kruskal–Wallis tests indicated that TS had significantly higher (*p* < 0.05) CF for the following elements: As compared with SP and NG2; Co and Cr compared with SP, PN, and MH; Ni compared with NG2; and Ba, Mn, and Sb compared with SP. In addition, NG1 had a significantly higher mean CF for Mn than SP, while both NG1 and LS had higher mean CF for Ba and Co compared with SP.

Consequently, all sampling points at TS recorded the highest PLI, ranging from 1 to 2, with a mean greater than 1, indicating roadside soil pollution (Fig. 4b, Table S6). In contrast, the PLI for all six sampling points at SP remained less than 1. At the remaining five sites, at least one sampling point showed PLI > 1; however, means remained less than 1 at all these sites (Fig. 4b). The mean PLI across the sites followed the order: TS > NG1 > LS > PN > NG2 > MH > SP. Site-wise comparisons revealed significantly higher (*p* < 0.05) mean PLI for TS compared with SP and MH, as well as for NG1 relative to SP.

This study reports notable multi-element roadside soil contamination across the investigated sites, thereby supporting the hypothesis that these often-overlooked traffic factors constitute significant sources of TE pollution. Although soil contamination typically ranged between low and moderate levels, more than half of the evaluated TEs (64%) were indicated as potential pollutants. Of all TEs, widespread contamination with Sb, Pb, Co, Cr, Mn, Ni, and Zn aligns well with global evidence showing the rising environmental occurrence of these elements near city traffic (Bukowiecki et al., 2009; Fussell et al., 2022; Sager, 2020). Roadside soil contamination with the emerging contaminant Sb is an important finding in this study. Although Sb is widely used in the automobile industry (Dousova et al., 2020), this TE has received limited research attention in non-metropolitan cities worldwide, which may result in the underestimation of pollution levels and associated monitoring and mitigation needs.

Compared to this study, previous investigations in South African cities recorded considerably elevated soil contamination with Pb and Cd adjacent to a national highway (Batwa Ismail et al., 2021) and with Cu, Ni, Pb, and Zn around gas stations in Pretoria (Olowoyo et al., 2022), which is understandable given the heavier traffic-related activities in these settings. On a global scale, relative to this study, generally higher soil contamination with TEs, including As, Cd, and Pb, were documented at traffic sites in capital and large cities worldwide, depending on prevailing factors like traffic intensity, climate, and land use (Kadi, 2009; Zhang et al., 2015; Pan et al., 2017; Li et al., 2022; Ayaz et al., 2023). Other international studies on smaller urban areas, such as one from India, documented TE pollution gradients more consistent with findings of this investigation (Adimalla et al., 2020). While another study from China showed partial similarity, with relatively higher Cd, Pb, and Zn contamination in soils around heavy traffic (Jiang et al., 2021). Likewise, an investigation near roads in Slovakia documented similar soil contamination by Cu and Zn, but relatively higher Cd and Pb pollution (Kuklová et al., 2022). Another study in an urban area in Poland reported considerable soil contamination with multiple common TEs, including Cu, Pb, and Zn, along highways (Grzebisz et al., 2002). However, the scarcity of published studies providing comprehensive assessments of multiple traffic conditions evaluated in this study, particularly in non-metropolitan cities, limits broader comparison with the present findings.

Although along with TS, NG1 and LS also showed higher contamination by individual TEs, TS emerged as the only site exhibiting soil contamination based on the cumulative elemental load. Moreover, the significant variation in pollution levels across sites and the exceedance of the safe PLI threshold at a minimum of one sampling point at all sites, except SP, are noteworthy. Overall, the spatial heterogeneity observed in urban soil pollution likely arises from the uneven distribution of surface pollutants driven by local extrinsic sources within these sites, consistent with previous studies (Ajmone-Marsan & Biasioli, 2010; Carrero et al., 2013; Hamzeh et al., 2011; Lee et al., 2021; Limo et al., 2018; Matzoros & Van Vliet, 1992). These patterns indicate the need for extended surveys to identify and prioritise localities such as TS that require monitoring.

## Risk assessment

### Ecological risk

The potential Er associated with evaluated TEs were all below 40, indicating low risk at present (Table 5, Table S7). Considering all sites, the largest Er ranges were recorded for Cd (2.75‒33.85), Pb (1.36‒31.68), As (3.32‒17.83), and Ni (3.64‒15.60). The RI for all seven sites, ranging between 25.98 and 72.42, reflected low risks (< 150) from cumulative elements (Table 5, Table S7). Across the seven localities, contributions of TEs to RI generally followed the order: Cd > Pb > As > Ni > Co > Cu > Cr > Zn > Mn. The site TS exhibited significantly higher (*p* < 0.05) mean Er for As, Co, Cr, and Mn compared with SP, for As and Ni compared to NG1, and for Co and Cr than PN and MH. In addition, NG2 showed a significantly higher mean Er for Mn compared to SP. In terms of RI, TS had a significantly higher mean relative to SP. Although the potential ecological risks from both individual and combined TEs are currently low at all sites, the substantial contributions of more hazardous Cd, Pb, As, and Ni to RI, together with Er values for Cd at SP and for Pb at MH approaching the moderate risk threshold of 40, at one sampling point each, indicate elevated future risks associated with continued accumulation.

**Table 5:** Potential ecological risk (Er) for trace elements in roadside soils and risk index (RI) for urban sites

Sampling sites		Potential ecological risk (Er)									Risk Index (RI)
		As	Cd	Co	Cr	Cu	Mn	Ni	Pb	Zn	
TS	Mean	13.28	6.54	8.67	5.29	5.04	1.94	9.37	5.89	2.22	58.24
	Max	17.83	7.41	9.54	6.13	6.05	2.38	15.60	6.91	2.97	67.03
	Min	9.58	5.44	7.04	4.59	3.70	1.60	6.95	4.93	1.00	49.64
	SD	3.31	0.71	0.90	0.53	0.77	0.33	3.18	0.77	0.69	5.66
LS	Mean	9.40	11.31	6.09	4.11	3.96	1.32	6.30	7.54	1.49	51.53
	Max	14.10	25.64	8.12	5.15	5.43	1.60	10.41	16.70	2.48	69.32
	Min	6.81	5.14	4.82	3.20	2.79	1.08	4.56	3.92	0.88	39.39
	SD	2.47	7.64	1.41	0.73	1.18	0.22	2.25	4.64	0.76	11.03
NG1	Mean	7.00	7.23	5.15	3.61	4.32	1.16	5.21	7.01	1.58	42.28
	Max	9.49	9.51	5.67	4.38	5.08	1.43	6.61	9.32	2.80	53.56
	Min	4.53	4.48	4.82	3.00	3.40	0.92	3.64	4.79	0.83	34.62
	SD	1.82	2.19	0.41	0.56	0.56	0.17	1.07	1.78	0.82	6.98
NG2	Mean	7.61	8.04	7.15	4.05	3.73	1.51	7.77	7.46	1.31	48.64
	Max	9.49	12.29	10.07	4.33	4.38	2.63	12.32	12.51	1.63	61.72
	Min	5.22	4.38	5.44	3.80	2.91	1.07	6.20	3.40	0.84	34.69
	SD	1.60	2.75	1.57	0.20	0.47	0.58	2.27	3.83	0.30	9.29
PN	Mean	7.68	8.16	5.06	3.32	5.42	1.19	7.09	4.57	1.42	43.92
	Max	9.44	23.06	6.01	3.63	15.54	1.69	8.03	6.76	1.93	59.03
	Min	6.31	3.86	4.72	2.95	3.16	0.97	5.41	3.64	1.06	35.43
	SD	1.33	7.51	0.47	0.28	4.96	0.26	1.19	1.12	0.29	10.85
SP	Mean	5.50	11.46	3.74	2.93	3.73	0.66	5.74	3.08	1.64	38.48
	Max	8.06	33.85	6.50	3.33	5.40	0.77	9.26	7.00	2.85	64.70
	Min	3.32	3.60	2.38	2.47	2.79	0.58	4.13	1.36	0.72	25.98
	SD	1.89	11.82	1.51	0.37	0.94	0.06	1.98	2.05	0.86	14.10
MH	Mean	8.44	5.21	5.09	3.33	4.30	1.18	5.43	8.49	1.26	42.72
	Max	10.46	9.48	5.72	3.88	6.07	1.68	8.05	31.68	1.89	72.42
	Min	5.92	3.28	4.03	2.93	3.04	0.87	4.35	3.00	1.00	33.95
	SD	1.56	2.28	0.67	0.31	1.33	0.31	1.33	11.39	0.32	14.82

Max, maximum; Min, minimum; SD, standard deviation (n = 6)

Globally, Cd has been recognised as a serious threat to urban ecosystems, as minor increases in environmental concentrations can exert far-reaching effects on biota, particularly edaphic biodiversity (Liao et al., 2022; Li et al., 2022; Suleymanov et al., 2025). The findings of this study correspond with global trends indicating As, Cd, Pb, and Ni as high-risk contaminants in urban soils in general, and in traffic-exposed soils in particular, across both capital and non-capital cities (Li et al., 2022; Poznanović Spahić et al., 2025; Suleymanov et al., 2025). Furthermore, consistent with contemporary literature, the present study suggests that elevated ecological risks generally relate to the extent of anthropogenic impact, particularly evident at TS in this city (Suleymanov et al., 2025).

### Human health risk

For both age groups, HQ for all evaluated TEs in roadside soils and corresponding HI remained far below the safe threshold of 1 through both exposure routes (Tables S8 and S9). Considering ingestion of soil-borne TEs, the mean HI across sites ranged as follows: adults, 0.0339–0.0774; children, 0.3163–0.7229. Via dermal contact, the mean HI ranged from 0.0393 to 0.1032 for adults and from 0.2573 to 0.6756 for children (Tables S8 and S9). 

**Fig. 5:**
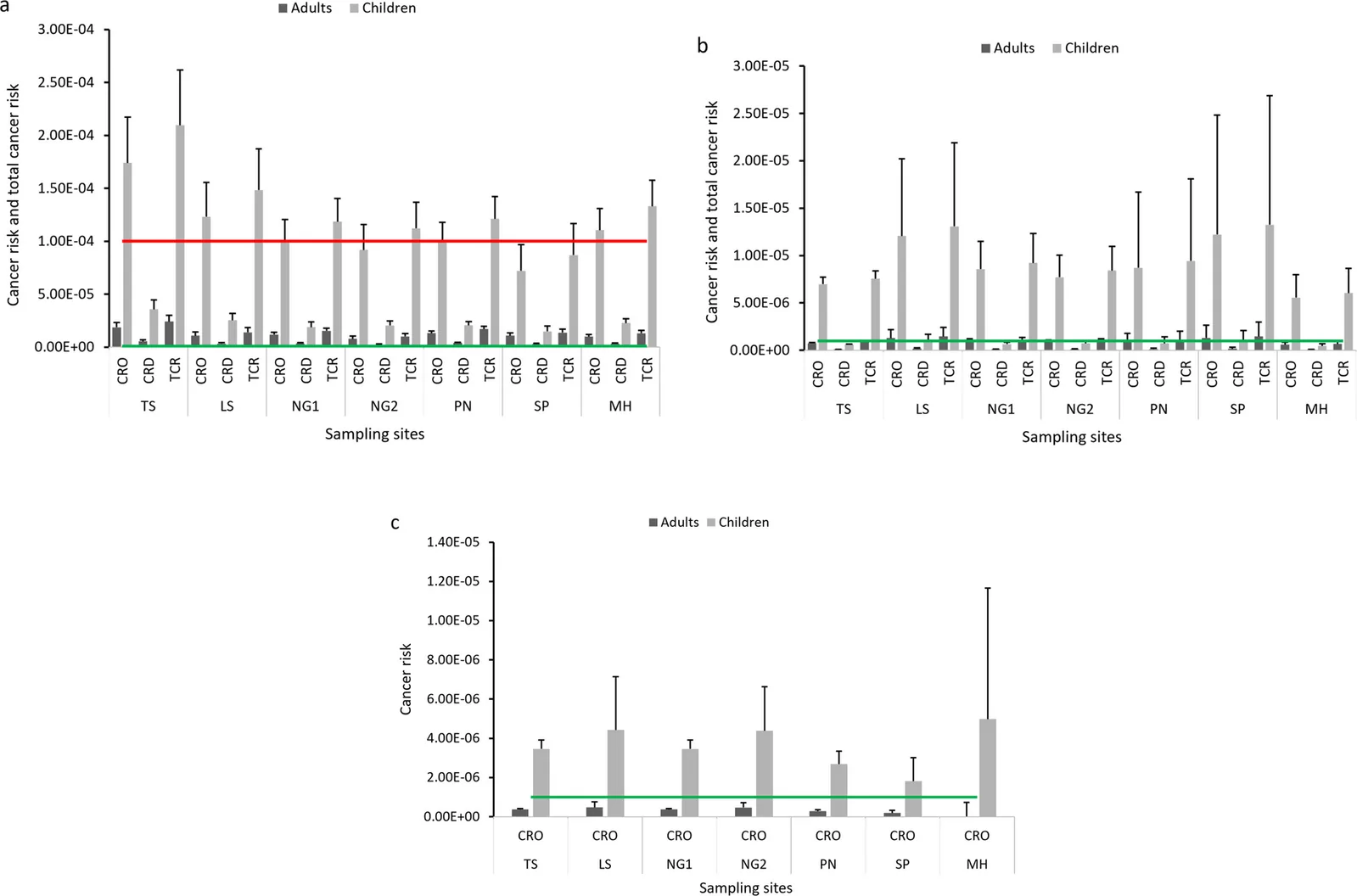
**a** Mean cancer risk (CR) and total cancer risk (TCR) for both age groups from arsenic, (**b**) mean CR and TCR for both age groups from cadmium, and **c** mean CR for both age groups from lead. CRO, cancer risk through ingestion; CRD, cancer risk through dermal exposure. CR and TCR thresholds: green line, 1 × 10^‒6^; red line, 1 × 10^‒4^. Error bars represent standard deviations (n = 6)

However, at multiple sites, As ingestion–driven CR and TCR for children exceeded the currently recommended unacceptable level of 1 × 10⁻^4^. In particular, at 57% of sites for CR, excluding NG1, NG2, and SP, and at 86% sites for TCR, excepting only SP (Fig. 5a). Conversely, the mean CR and TCR linked to As via ingestion of roadside soils stayed between 1 × 10⁻^6^ and 1 × 10⁻^4^ for adults (Fig. 5a, Table S10), which is acceptable currently. Similarly, dermal exposure to As could be of no concern for both age groups (Fig. 5a). In comparison with As, Cd exhibited lower mean CR and TCR for both age groups and exposure routes (Fig. 5b). A similar pattern was observed for Pb via the ingestion pathway (Fig. 5c). For both Cd and Pb, risks generally remained below 1 × 10⁻⁶ and, in some cases, fell within the range of 1 × 10⁻⁶ to 1 × 10⁻^4^ (Fig. 5b, c). Kruskal–Wallis tests indicated significant differences (*p* < 0.05) in mean CR associated with As between TS and SP across both exposure pathways and age groups. In addition, for both age groups, significant differences in mean CR were observed between TS and NG2 for As ingestion. Mean TCR for As also differed significantly between TS and SP, as well as between TS and NG2. The site TS exhibited higher means in all the above comparisons. Mann–Whitney U tests indicated significantly higher risks (*p* < 0.05) for children than for adults through both exposure routes.

The results suggest that there are currently no apparent non-carcinogenic risks to either age group from the assessed TEs in roadside soils at the investigated sites. However, roadside soil As-driven CR through ingestion, along with TCR, appears to be concerning for children, particularly at the school site TS. Consistent with global observations, children exhibit heightened susceptibility to TEs relative to adults, and ingestion represents the major health-threatening pathway compared with dermal exposure in this study (Li et al., 2018; Li et al., 2022; Olowoyo et al., 2022; Pan et al., 2017). Studies by Batwa-Ismail et al. (2021) and Olowoyo et al. (2022) reported higher health risks to both age groups from multiple TEs, including As, in South African localities characterised by heavier traffic than the studied sites. The findings of this study follow the global trend of As being among the chief TEs driving public health risks in both smaller and larger cities worldwide (De Miguel et al., 2017; Pan et al., 2017).

The IARC has classified all As compounds as human carcinogens (IARC, 2012), and several As formations are also recognised as teratogenic and mutagenic, making them among the most dangerous environmental contaminants (Cai & Zhang, 2021; Irwin et al., 2025). Its cytotoxicity primarily results from the generation of reactive oxygen species, depletion of antioxidants, DNA damage, and initiation of malignant growth (Carver & Gallicchio, 2017; Dousova et al., 2020). Exposure to As has been linked to lung, bladder, colon, and skin cancers, and even low-dose exposure has been associated with pancreatic cancer (Carver & Gallicchio, 2017).

Nevertheless, the estimated pollution levels and ecological and human health risks reported in this study should be interpreted with caution, as total concentration based assessments tend to present overestimated values. Therefore, such assessments using bioavailable fractions are recommended to generate more realistic scenarios and select the most effective mitigation measures (Sutherland, 2002; Yang et al., 2022). This is particularly important given that the oral bioavailability of As varies substantially among sites depending on soil properties and the presence of chemical formations, and is generally much lower than that of its water soluble forms (US Epa, 2012; Bradham et al., 2018; Irwin et al., 2025). However, the findings of this study provide baseline data for future investigations and recommend the examination of diverse environmental compartments, including watercourses within the city, to provide more comprehensive insights into the risks associated with traffic-emitted elements.

Concurrently, research on cost effective mitigation strategies, such as the establishment of roadside hedgerows and the application of mulch and compost to roadside soils, is essential, as these measures can simultaneously address multiple challenges prevalent in growing cities worldwide, including air pollution, human exposure to particulate matter, soil degradation and erosion, and the decline of edaphic biodiversity (US Epa, 1999; Weindorf et al., 2013; Biocca et al., 2024). In addition, the construction of road shoulders or pavements may further help to suppress hazardous particle generation and resuspension. Finally, the development of well-planned pedestrian and cycling infrastructure (Okosun et al., 2023) could offer sustainable long-term measures to reduce traffic burden and related pollution in small and medium-sized cities.

## Conclusions

This study explored the influence of multiple often overlooked but prevalent traffic conditions in non-metropolitan cities globally on TE contamination of roadside soils and associated risks in a secondary South African city. The findings indicate that Cu and Pb may act as soil-degrading TEs, while Sb, together with Cr, Co, Mn, Ni, and Zn, pose additional environmental risks. This finding emphasises the need to consider Sb, an emerging contaminant, in traffic-related pollution and risk assessments in relatively small cities. While road proximity emerges as the primary traffic-associated contributor to TE contamination in roadside soils, proximity to a highway, roadside transport stop, and parking area also represents notable sources. These findings underline that the evaluation of the pollution footprint of transport stops, a common yet underexamined traffic factor in many cities of developing countries, should be prioritised in future research. The presence of multiple traffic-origin points for most TEs indicates that locality-specific traffic gradients collectively drive roadside soil pollution patterns across urban zones. Consequently, sites influenced by several traffic conditions may function as pollution hotspots, necessitating monitoring and targeted traffic management. Although the overall ecological risk remains low, the relatively greater contributions of Cd, As, Pb, and Ni should not be overlooked, particularly under continued accumulation in roadside soils. Furthermore, the potential cancer risk to children from exposure to As in roadside soils requires follow-up investigation. Therefore, under comparable traffic patterns, travellers should avoid waiting near road edges to reduce TE exposure. Compost application, establishment of roadside hedgerows, and relocation of transport stops may represent affordable strategies for mitigating TE pollution at these roadside facilities. However, further research is needed to identify suitable hedgerow species and determine the most appropriate compost types for the study area. In addition, it is imperative to consider broader spatial gradients and soil depths, as well as to assess potential contamination across interconnected environmental compartments, particularly water systems, to inform a comprehensive mitigation approach in similar urban settings. Integrating multiple locality-specific traffic conditions into soil pollution and risk assessments could therefore improve pollution management and reinforce the importance of addressing traffic-related challenges to enhance ecosystem sustainability in comparable cities.

## Supplementary Information

Below is the link to the electronic supplementary material.

ESM 1 (DOCX 108 KB)

## Data Availability

All data supporting the findings of this study are available within the paper and its Supplementary Information.
