# Supplementary material for: Evaluating the pollution footprint of traffic-derived trace elements in roadside soil and the associated risks in a non-metropolitan city
Source: Environ Monit Assess. 2026 Aug 6;198(9):919. doi: 10.1007/s10661-026-15727-x (PMC13447398; doi:10.1007/s10661-026-15727-x)
Supplement: Supplementary file 1 [file 10661_2026_15727_MOESM1_ESM.docx]

**Supplementary Information**

**Evaluating the pollution footprint of traffic-derived trace elements in roadside soil and the associated risks in a non-metropolitan city**

Sutapa Adhikari

Unit for Environmental Sciences and Management, North-West University. Potchefstroom, 2531, South Africa South African Environmental Observation Network, Ndlovu Node, WITS Rural Campus, Acornhoek, 1360, South Africa

Table S1. Pollution and ecological risk categories.

| Pollution index | Pollution classes | Reference |
| --- | --- | --- |
| Index of geo-accumulation (I_geo_) | Igeo ≤ 0, Uncontaminated \| \|; 0 < Igeo ≤ 1, Uncontaminated to moderately contaminated \| \|; 1 < Igeo ≤ 2, Moderately contaminated \| \|; 2 < Igeo ≤ 3, Moderately to heavily contaminated \| \|; 3 < Igeo ≤ 4, Heavily contaminated \| \|; 4 < Igeo ≤ 5, Heavily to extremely contaminated \| \|; Igeo > 5, Extremely contaminated \| \| | Müller, 1969 |
| Contamination factor (CF) | CF < 1, Low contamination \|; 1 ≤ CF < 3, Moderate contamination \|; 3 ≤ CF < 6, Considerable contamination \|; CF ≥ 6, Very high contamination \| | Håkanson, 1980 |
| Pollution load index (PLI) | PLI < 1, No contamination  PLI 1‒2, Medium contamination  PLI 2‒5, Heavy contamination  PLI > 5, Extreme contamination | Jiang et al., 2021 |
| Ecological risk (Er) | Er < 40, Low ecological risk  40 ≤ Er < 80, Moderate ecological risk  80 ≤ Er < 160, Considerable ecological risk  160 ≤ Er < 320, High ecological risk  Er ≥ 320, Very high ecological risk. | Håkanson, 1980 |
| Ecological risk index (RI) | RI < 150, Low ecological risk  150 ≤ RI < 300, Moderate ecological risk  300 ≤ RI < 600, Considerable ecological risk  RI ≥ 600, Very high ecological risk | Håkanson, 1980 |

Table S2. Values of parameters used to calculate non-carcinogenic risk (DEA, 2010).

| Parameters | Children | Adults |
| --- | --- | --- |
| Ingestion rate (IngR, mg/kg-day) | 200 | 100 |
| Exposure factor (EF, day/year) | 350 | 350 |
| Exposure duration (ED, years) | 6 | 70 |
| Average time (AT, days) | 2190 | 25550 |
| Body weight (BW, kg) | 15 | 70 |
| Skin/dermal surface area (SA, cm^2^) | 2100 | 5800 |
| Adherence factor (AF, mg/cm^2^) | 0.2 | 0.07 |
| Conversion factor (CF, kg/mg) | 1×10^−6^ | 1×10^−6^ |
| Dermal absorption efficiency (ABS) | 0.03 for As; 0.001 for other elements (for both age groups) | |

Table S3. Reference dose (RfD) and cancer slope factor (SF) for evaluated trace elements.

| Values (mg/kg-day) | Oral | Dermal | References |
| --- | --- | --- | --- |
| RfD | RfD_O_  As: 0.0003; Ba: 0.2; Cd: 0.001; Co: 0.03; Cr: 1.5; Cu: 0.04; Mn: 0.14; Ni: 0.02; Pb: 0.0035; Sb: 0.0004; Zn: 0.3 | RfD_ABS_  As: 0.000123; Ba: 2.9; Cd: 0.00001; Co: 0.016; Cr: 0.0195; Cu: 0.012; Mn: 0.00184; Ni: 0.0054; Pb: 0.000525; Sb: 0.0027; Zn: 0.06 | Finley et al., 2012; IRIS, 1989; IRIS, 1991; IRIS, 2004; IRIS, 2005; US EPA, 2002; Relić et al., 2019; Rinklebe et al., 2019; Miletić et al., 2023 |
| SF | SF_O_  As: 1.5; Cd: 6.1; Pb: 0.0085 | SF_ABS_  As: 3.66; Cd: 6.1. | Timofeev et al., 2019; US EPA, 2002; Miletić et al., 2023 |

Table S4. Concentrations of trace elements at six sampling points (SP1‒SP6) in each locality.

| Sites | Trace elements | | | | | | | | | | |
| --- | --- | --- | --- | --- | --- | --- | --- | --- | --- | --- | --- |
|  | As | Ba | Cd | Co | Cr | Cu | Mn | Ni | Pb | Sb | Zn |
| **TS** |  |  |  |  |  |  |  |  |  |  |  |
| SP1 | 9.51 | 198.34 | 0.09 | 18.94 | 182.29 | 40.75 | 860.16 | 90.50 | 32.98 | 2.54 | 207.83 |
| SP2 | 8.61 | 198.71 | 0.10 | 20.37 | 157.66 | 40.69 | 925.72 | 55.31 | 33.21 | 1.75 | 178.75 |
| SP3 | 10.90 | 138.41 | 0.08 | 19.58 | 145.14 | 28.80 | 1124.89 | 40.32 | 37.30 | 0.90 | 70.26 |
| SP4 | 12.18 | 196.28 | 0.07 | 21.15 | 164.73 | 37.96 | 781.52 | 48.82 | 27.10 | 1.81 | 137.05 |
| SP5 | 6.54 | 237.39 | 0.10 | 21.56 | 156.96 | 47.10 | 1162.00 | 47.31 | 33.73 | 2.00 | 155.31 |
| SP6 | 6.69 | 142.77 | 0.09 | 15.91 | 136.60 | 39.99 | 824.53 | 43.74 | 26.60 | 1.70 | 182.99 |
| **LS** |  |  |  |  |  |  |  |  |  |  |  |
| SP1 | 6.29 | 145.31 | 0.09 | 17.01 | 136.48 | 23.72 | 760.35 | 35.15 | 29.97 | 0.87 | 61.43 |
| SP2 | 5.68 | 93.28 | 0.15 | 11.14 | 127.99 | 31.66 | 527.11 | 27.36 | 41.43 | 1.22 | 172.56 |
| SP3 | 4.65 | 113.67 | 0.35 | 10.90 | 95.05 | 24.24 | 554.53 | 60.35 | 21.18 | 1.02 | 69.72 |
| SP4 | 6.42 | 142.74 | 0.18 | 18.35 | 153.27 | 42.27 | 782.54 | 41.73 | 90.17 | 1.09 | 173.41 |
| SP5 | 5.86 | 110.99 | 0.07 | 13.40 | 119.40 | 21.73 | 652.66 | 28.24 | 30.53 | 0.58 | 74.57 |
| SP6 | 9.63 | 124.84 | 0.09 | 11.83 | 102.24 | 41.33 | 584.48 | 26.46 | 30.89 | 0.75 | 74.09 |
| **NG1** |  |  |  |  |  |  |  |  |  |  |  |
| SP1 | 6.03 | 125.27 | 0.08 | 14.30 | 123.36 | 28.72 | 741.20 | 41.11 | 25.28 | 1.22 | 113.97 |
| SP2 | 6.48 | 95.35 | 0.11 | 15.64 | 124.95 | 29.56 | 583.94 | 42.18 | 28.80 | 0.80 | 108.89 |
| SP3 | 3.57 | 127.86 | 0.06 | 15.31 | 112.96 | 22.68 | 562.61 | 35.98 | 18.36 | 0.50 | 58.74 |
| SP4 | 5.51 | 157.53 | 0.12 | 16.72 | 128.74 | 29.88 | 730.01 | 42.43 | 67.55 | 1.99 | 101.51 |
| SP5 | 5.33 | 89.89 | 0.17 | 12.29 | 116.72 | 29.17 | 521.06 | 37.24 | 37.86 | 0.88 | 90.11 |
| SP6 | 4.26 | 266.41 | 0.13 | 22.75 | 116.49 | 34.05 | 1283.69 | 71.44 | 63.84 | 0.78 | 77.83 |
| **NG2** |  |  |  |  |  |  |  |  |  |  |  |
| SP1 | 3.63 | 105.14 | 0.06 | 10.89 | 130.23 | 26.46 | 584.62 | 21.11 | 35.40 | 0.88 | 58.43 |
| SP2 | 5.08 | 128.27 | 0.08 | 11.10 | 114.32 | 31.80 | 596.64 | 25.25 | 34.35 | 0.62 | 66.00 |
| SP3 | 3.09 | 95.63 | 0.08 | 11.05 | 89.39 | 33.09 | 446.70 | 33.95 | 25.86 | 0.70 | 195.71 |
| SP4 | 4.86 | 117.27 | 0.12 | 12.82 | 96.18 | 34.57 | 552.10 | 32.12 | 48.73 | 1.32 | 85.28 |
| SP5 | 5.56 | 86.07 | 0.13 | 11.12 | 93.64 | 36.13 | 533.42 | 30.60 | 32.59 | 0.68 | 88.16 |
| SP6 | 6.48 | 100.08 | 0.13 | 12.81 | 120.41 | 39.50 | 697.25 | 38.31 | 50.33 | 1.32 | 168.59 |
| **PN** |  |  |  |  |  |  |  |  |  |  |  |
| SP1 | 4.58 | 99.91 | 0.06 | 11.03 | 90.75 | 25.79 | 530.08 | 46.57 | 23.50 | 0.74 | 90.59 |
| SP2 | 4.48 | 93.08 | 0.32 | 10.91 | 105.17 | 27.83 | 471.72 | 46.58 | 24.65 | 0.62 | 101.03 |
| SP3 | 6.03 | 120.60 | 0.05 | 11.26 | 108.06 | 26.53 | 603.85 | 46.38 | 21.51 | 1.06 | 90.80 |
| SP4 | 4.31 | 107.01 | 0.07 | 10.66 | 104.79 | 27.23 | 524.22 | 42.00 | 19.65 | 1.01 | 105.91 |
| SP5 | 6.45 | 148.14 | 0.12 | 13.58 | 95.86 | 120.93 | 826.26 | 33.77 | 36.48 | 1.36 | 134.91 |
| SP6 | 5.63 | 103.61 | 0.06 | 11.23 | 87.84 | 24.62 | 541.24 | 31.39 | 22.40 | 2.03 | 74.10 |
| **SP** |  |  |  |  |  |  |  |  |  |  |  |
| SP1 | 2.61 | 52.59 | 0.05 | 5.76 | 90.98 | 30.44 | 283.36 | 26.37 | 7.32 | 0.48 | 165.89 |
| SP2 | 4.63 | 57.91 | 0.06 | 7.16 | 73.35 | 29.16 | 314.79 | 23.95 | 37.80 | 1.16 | 101.60 |
| SP3 | 4.48 | 54.31 | 0.21 | 9.26 | 99.04 | 22.39 | 311.77 | 40.06 | 11.24 | 0.41 | 50.30 |
| SP4 | 3.07 | 54.11 | 0.46 | 14.69 | 93.76 | 21.68 | 317.16 | 53.72 | 17.59 | 0.58 | 52.60 |
| SP5 | 2.27 | 48.64 | 0.05 | 5.38 | 92.35 | 42.00 | 321.35 | 29.55 | 9.94 | 0.50 | 199.53 |
| SP6 | 5.51 | 68.01 | 0.11 | 8.40 | 73.41 | 28.27 | 377.61 | 26.23 | 16.02 | 0.74 | 117.99 |
| **MH** |  |  |  |  |  |  |  |  |  |  |  |
| SP1 | 6.18 | 65.41 | 0.05 | 9.11 | 97.78 | 47.23 | 484.84 | 25.22 | 16.20 | 0.75 | 83.77 |
| SP2 | 4.05 | 71.93 | 0.04 | 12.92 | 97.72 | 25.73 | 422.42 | 46.69 | 16.35 | 0.81 | 75.40 |
| SP3 | 6.08 | 98.92 | 0.13 | 10.82 | 115.35 | 42.38 | 449.21 | 31.43 | 171.09 | 3.38 | 132.51 |
| SP4 | 5.09 | 112.87 | 0.05 | 10.81 | 87.11 | 24.03 | 643.07 | 29.84 | 23.35 | 0.65 | 70.09 |
| SP5 | 6.02 | 115.06 | 0.08 | 12.67 | 96.55 | 23.66 | 624.37 | 27.35 | 21.64 | 0.61 | 80.80 |
| SP6 | 7.15 | 118.75 | 0.07 | 12.65 | 99.49 | 37.89 | 818.76 | 28.43 | 26.42 | 1.01 | 85.21 |

Table S5. Index of geo-accumulation for soil trace elements at six sampling points (SP1‒SP6) across sites.

| TS |  | As | Ba | Cd | Co | Cr | Cu | Mn | Ni | Pb | Sb | Zn |
| --- | --- | --- | --- | --- | --- | --- | --- | --- | --- | --- | --- | --- |
|  | SP1 | 0.402 | -2.239 | -0.515 | -0.454 | 0.402 | -0.044 | -0.650 | 0.360 | 0.371 | 2.080 | 1.048 |
|  | SP2 | 0.258 | -2.236 | -0.494 | -0.349 | 0.192 | -0.046 | -0.544 | -0.350 | 0.381 | 1.547 | 0.831 |
|  | SP3 | 0.598 | -2.758 | -0.674 | -0.407 | 0.073 | -0.544 | -0.263 | -0.806 | 0.549 | 0.584 | -0.516 |
|  | SP4 | 0.759 | -2.254 | -0.861 | -0.295 | 0.255 | -0.146 | -0.789 | -0.530 | 0.088 | 1.591 | 0.448 |
|  | SP5 | -0.138 | -1.979 | -0.415 | -0.268 | 0.186 | 0.165 | -0.216 | -0.575 | 0.404 | 1.737 | 0.628 |
|  | SP6 | -0.106 | -2.713 | -0.649 | -0.706 | -0.015 | -0.071 | -0.711 | -0.689 | 0.061 | 1.505 | 0.865 |
| LS | SP1 | -0.196 | -2.687 | -0.555 | -0.610 | -0.016 | -0.824 | -0.828 | -1.004 | 0.233 | 0.542 | -0.710 |
|  | SP2 | -0.342 | -3.327 | 0.108 | -1.220 | -0.109 | -0.408 | -1.357 | -1.366 | 0.700 | 1.029 | 0.780 |
|  | SP3 | -0.629 | -3.042 | 1.376 | -1.251 | -0.538 | -0.793 | -1.284 | -0.224 | -0.268 | 0.759 | -0.528 |
|  | SP4 | -0.166 | -2.713 | 0.423 | -0.500 | 0.151 | 0.009 | -0.787 | -0.757 | 1.822 | 0.861 | 0.787 |
|  | SP5 | -0.296 | -3.076 | -0.942 | -0.954 | -0.209 | -0.951 | -1.049 | -1.320 | 0.260 | -0.040 | -0.431 |
|  | SP6 | 0.419 | -2.906 | -0.605 | -1.133 | -0.433 | -0.023 | -1.208 | -1.414 | 0.277 | 0.318 | -0.440 |
| NG1 | SP1 | -0.255 | -2.901 | -0.733 | -0.860 | -0.162 | -0.549 | -0.865 | -0.778 | -0.012 | 1.022 | 0.181 |
|  | SP2 | -0.151 | -3.295 | -0.357 | -0.731 | -0.143 | -0.507 | -1.209 | -0.741 | 0.176 | 0.422 | 0.116 |
|  | SP3 | -1.013 | -2.872 | -1.173 | -0.761 | -0.289 | -0.889 | -1.263 | -0.970 | -0.474 | -0.268 | -0.775 |
|  | SP4 | -0.386 | -2.571 | -0.186 | -0.634 | -0.100 | -0.491 | -0.887 | -0.732 | 1.405 | 1.730 | 0.014 |
|  | SP5 | -0.435 | -3.380 | 0.315 | -1.078 | -0.242 | -0.526 | -1.373 | -0.921 | 0.570 | 0.551 | -0.157 |
|  | SP6 | -0.756 | -1.813 | -0.097 | -0.190 | -0.244 | -0.303 | -0.073 | 0.019 | 1.324 | 0.378 | -0.369 |
| NG2 | SP1 | -0.988 | -3.154 | -1.142 | -1.252 | -0.084 | -0.666 | -1.207 | -1.740 | 0.473 | 0.553 | -0.782 |
|  | SP2 | -0.503 | -2.867 | -0.823 | -1.225 | -0.272 | -0.402 | -1.178 | -1.481 | 0.430 | 0.037 | -0.607 |
|  | SP3 | -1.218 | -3.291 | -0.759 | -1.232 | -0.627 | -0.344 | -1.596 | -1.054 | 0.020 | 0.231 | 0.962 |
|  | SP4 | -0.567 | -2.997 | -0.209 | -1.017 | -0.521 | -0.281 | -1.290 | -1.134 | 0.934 | 1.136 | -0.237 |
|  | SP5 | -0.372 | -3.443 | -0.066 | -1.223 | -0.559 | -0.217 | -1.340 | -1.204 | 0.354 | 0.182 | -0.189 |
|  | SP6 | -0.152 | -3.225 | -0.054 | -1.018 | -0.197 | -0.089 | -0.953 | -0.880 | 0.981 | 1.134 | 0.746 |
| PN | SP1 | -0.653 | -3.228 | -1.225 | -1.235 | -0.605 | -0.704 | -1.349 | -0.598 | -0.118 | 0.294 | -0.150 |
|  | SP2 | -0.683 | -3.330 | 1.223 | -1.250 | -0.392 | -0.594 | -1.517 | -0.598 | -0.049 | 0.043 | 0.008 |
|  | SP3 | -0.255 | -2.956 | -1.354 | -1.204 | -0.353 | -0.663 | -1.161 | -0.604 | -0.246 | 0.816 | -0.146 |
|  | SP4 | -0.741 | -3.129 | -1.035 | -1.283 | -0.397 | -0.625 | -1.365 | -0.747 | -0.376 | 0.754 | 0.076 |
|  | SP5 | -0.159 | -2.660 | -0.197 | -0.934 | -0.526 | 1.526 | -0.708 | -1.062 | 0.517 | 1.180 | 0.425 |
|  | SP6 | -0.356 | -3.175 | -1.174 | -1.208 | -0.652 | -0.771 | -1.319 | -1.168 | -0.187 | 1.757 | -0.440 |
| SP | SP1 | -1.467 | -4.154 | -1.382 | -2.172 | -0.601 | -0.464 | -2.252 | -1.419 | -1.800 | -0.324 | 0.723 |
|  | SP2 | -0.638 | -4.015 | -1.130 | -1.857 | -0.912 | -0.527 | -2.101 | -1.558 | 0.568 | 0.949 | 0.016 |
|  | SP3 | -0.683 | -4.107 | 0.629 | -1.487 | -0.479 | -0.907 | -2.114 | -0.816 | -1.182 | -0.562 | -0.998 |
|  | SP4 | -1.231 | -4.112 | 1.777 | -0.821 | -0.558 | -0.954 | -2.090 | -0.392 | -0.535 | -0.060 | -0.934 |
|  | SP5 | -1.666 | -4.266 | -1.455 | -2.270 | -0.579 | 0.000 | -2.071 | -1.255 | -1.359 | -0.252 | 0.989 |
|  | SP6 | -0.387 | -3.783 | -0.358 | -1.627 | -0.911 | -0.571 | -1.838 | -1.426 | -0.671 | 0.308 | 0.232 |
| MH | SP1 | -0.220 | -3.839 | -1.340 | -1.510 | -0.497 | 0.169 | -1.477 | -1.483 | -0.654 | 0.326 | -0.263 |
|  | SP2 | -0.832 | -3.702 | -1.592 | -1.006 | -0.498 | -0.707 | -1.676 | -0.594 | -0.641 | 0.427 | -0.414 |
|  | SP3 | -0.243 | -3.242 | -0.060 | -1.262 | -0.259 | 0.013 | -1.587 | -1.165 | 2.746 | 2.495 | 0.399 |
|  | SP4 | -0.499 | -3.052 | -1.338 | -1.264 | -0.664 | -0.806 | -1.070 | -1.241 | -0.127 | 0.124 | -0.520 |
|  | SP5 | -0.258 | -3.024 | -0.747 | -1.035 | -0.515 | -0.828 | -1.112 | -1.366 | -0.237 | 0.014 | -0.315 |
|  | SP6 | -0.011 | -2.979 | -1.036 | -1.037 | -0.472 | -0.149 | -0.721 | -1.310 | 0.051 | 0.758 | -0.238 |

Table S6. Contamination factor (CF) and pollution load index (PLI) values for six sampling points (SP1‒SP6) across sites.

|  | Sampling points | | | | | | |
| --- | --- | --- | --- | --- | --- | --- | --- |
| **TS** | SP1 | SP2 | SP3 | SP4 | SP5 | SP6 | Mean |
| As | 1.392886 | 1.260501 | 1.595391 | 1.783384 | 0.957794 | 0.979230 | 1.328197 |
| Ba | 0.431172 | 0.431983 | 0.300886 | 0.426690 | 0.516076 | 0.310360 | 0.402861 |
| Cd | 0.230488 | 0.233756 | 0.206341 | 0.181293 | 0.246976 | 0.209976 | 0.218138 |
| Co | 1.676127 | 1.802926 | 1.732468 | 1.871535 | 1.907725 | 1.408092 | 1.733145 |
| Cr | 3.063729 | 2.649761 | 2.439336 | 2.768649 | 2.637925 | 2.295808 | 2.642535 |
| Cu | 1.04749 | 1.046033 | 0.740430 | 0.975841 | 1.210743 | 1.028021 | 1.008093 |
| Mn | 1.76262 | 1.896968 | 2.305102 | 1.601478 | 2.381142 | 1.689610 | 1.939487 |
| Ni | 3.120659 | 1.907133 | 1.390338 | 1.683588 | 1.631506 | 1.508340 | 1.873594 |
| Pb | 1.221563 | 1.229838 | 1.381579 | 1.003868 | 1.249319 | 0.985353 | 1.178587 |
| Sb | 3.787209 | 2.617537 | 1.342821 | 2.697507 | 2.985791 | 2.540836 | 2.661950 |
| Zn | 2.969008 | 2.553586 | 1.003677 | 1.957898 | 2.218644 | 2.614147 | 2.219493 |
| **PLI** | 1.442469 | 1.306399 | 1.057498 | 1.232529 | 1.334545 | 1.121707 | 1.249191 |
| **LS** | SP1 | SP2 | SP3 | SP4 | SP5 | SP6 | Mean |
| As | 0.920366 | 0.831958 | 0.681422 | 0.939425 | 0.85869 | 1.409537 | 0.940233 |
| Ba | 0.315899 | 0.202775 | 0.247102 | 0.310306 | 0.241273 | 0.271395 | 0.264792 |
| Cd | 0.224073 | 0.354756 | 0.854707 | 0.441537 | 0.171390 | 0.216512 | 0.377163 |
| Co | 1.504965 | 0.985516 | 0.964946 | 1.623970 | 1.185531 | 1.047112 | 1.218673 |
| Cr | 2.293837 | 2.151160 | 1.597556 | 2.575989 | 2.006765 | 1.718324 | 2.057272 |
| Cu | 0.609714 | 0.813785 | 0.623066 | 1.086603 | 0.558548 | 1.062532 | 0.792375 |
| Mn | 1.558102 | 1.080143 | 1.136341 | 1.603575 | 1.33741 | 1.197697 | 1.318878 |
| Ni | 1.212195 | 0.943461 | 2.081202 | 1.438847 | 0.973676 | 0.912424 | 1.260301 |
| Pb | 1.109859 | 1.534265 | 0.784260 | 3.339781 | 1.130560 | 1.144149 | 1.507146 |
| Sb | 1.303552 | 1.827090 | 1.516000 | 1.626627 | 0.871328 | 1.116493 | 1.376848 |
| Zn | 0.877528 | 2.465202 | 0.995934 | 2.477268 | 1.065271 | 1.058410 | 1.489935 |
| **PLI** | 0.903827 | 0.971447 | 0.917336 | 1.301611 | 0.779408 | 0.876292 | 0.95832 |
| **NG1** | SP1 | SP2 | SP3 | SP4 | SP5 | SP6 | Mean |
| As | 0.883256 | 0.949145 | 0.522444 | 0.806568 | 0.77976 | 0.624004 | 0.760863 |
| Ba | 0.272328 | 0.207293 | 0.277947 | 0.342466 | 0.195404 | 0.579159 | 0.312433 |
| Cd | 0.198122 | 0.257049 | 0.146049 | 0.289341 | 0.409683 | 0.307878 | 0.268020 |
| Co | 1.265530 | 1.383963 | 1.354858 | 1.480028 | 1.087489 | 2.013516 | 1.430897 |
| Cr | 2.073244 | 2.099945 | 1.898520 | 2.163675 | 1.961619 | 1.957776 | 2.025796 |
| Cu | 0.738204 | 0.760006 | 0.582953 | 0.768179 | 0.749864 | 0.875407 | 0.745769 |
| Mn | 1.518853 | 1.196606 | 1.152887 | 1.495928 | 1.067749 | 2.630516 | 1.510423 |
| Ni | 1.417540 | 1.454592 | 1.240657 | 1.463200 | 1.284220 | 2.463477 | 1.553948 |
| Pb | 0.936436 | 1.066698 | 0.679955 | 2.501777 | 1.402305 | 2.364353 | 1.491921 |
| Sb | 1.819075 | 1.199836 | 0.743672 | 2.970955 | 1.312000 | 1.163925 | 1.534910 |
| Zn | 1.628138 | 1.555619 | 0.839138 | 1.450214 | 1.287283 | 1.111843 | 1.312039 |
| **PLI** | 0.947284 | 0.917342 | 0.698463 | 1.149689 | 0.902690 | 1.202658 | 0.969688 |
| **NG2** | SP1 | SP2 | SP3 | SP4 | SP5 | SP6 | Mean |
| As | 0.531461 | 0.743855 | 0.453054 | 0.71157 | 0.814417 | 0.948533 | 0.700482 |
| Ba | 0.228570 | 0.278855 | 0.207892 | 0.254932 | 0.187100 | 0.217556 | 0.229151 |
| Cd | 0.149220 | 0.186073 | 0.194585 | 0.284927 | 0.314463 | 0.317098 | 0.241061 |
| Co | 0.964108 | 0.982193 | 0.977842 | 1.134559 | 0.983688 | 1.13395 | 1.02939 |
| Cr | 2.18875 | 1.921273 | 1.502283 | 1.616482 | 1.573839 | 2.023753 | 1.804397 |
| Cu | 0.680325 | 0.817401 | 0.850748 | 0.88868 | 0.928763 | 1.015417 | 0.863556 |
| Mn | 1.197986 | 1.222625 | 0.915361 | 1.131355 | 1.093071 | 1.428791 | 1.164865 |
| Ni | 0.727875 | 0.870691 | 1.170772 | 1.107647 | 1.055056 | 1.321086 | 1.042188 |
| Pb | 1.311263 | 1.272340 | 0.957710 | 1.804769 | 1.206930 | 1.864165 | 1.402863 |
| Sb | 1.314179 | 0.918552 | 1.051045 | 1.968194 | 1.015866 | 1.965537 | 1.372229 |
| Zn | 0.834758 | 0.942868 | 2.795892 | 1.218307 | 1.259493 | 2.408461 | 1.57663 |
| **PLI** | 0.732635 | 0.785086 | 0.784309 | 0.931257 | 0.826358 | 1.088433 | 0.858013 |
| **PN** | SP1 | SP2 | SP3 | SP4 | SP5 | SP6 | Mean |
| As | 0.670568 | 0.656660 | 0.883211 | 0.630549 | 0.944098 | 0.823848 | 0.768156 |
| Ba | 0.217190 | 0.202340 | 0.262170 | 0.232633 | 0.322050 | 0.225239 | 0.243604 |
| Cd | 0.140878 | 0.768805 | 0.128780 | 0.160659 | 0.287171 | 0.145976 | 0.272045 |
| Co | 0.975710 | 0.965253 | 0.996500 | 0.943691 | 1.201581 | 0.993933 | 1.012778 |
| Cr | 1.525201 | 1.767517 | 1.816212 | 1.761234 | 1.611042 | 1.476291 | 1.659583 |
| Cu | 0.662856 | 0.715469 | 0.682097 | 0.699989 | 3.108832 | 0.632898 | 1.08369 |
| Mn | 1.086230 | 0.966645 | 1.237389 | 1.074225 | 1.693154 | 1.109090 | 1.194455 |
| Ni | 1.606007 | 1.606260 | 1.599419 | 1.448177 | 1.164322 | 1.082274 | 1.417743 |
| Pb | 0.870255 | 0.913116 | 0.796587 | 0.727763 | 1.351200 | 0.829493 | 0.914736 |
| Sb | 1.097925 | 0.922552 | 1.576776 | 1.509836 | 2.029701 | 3.027224 | 1.694002 |
| Zn | 1.294209 | 1.443250 | 1.297153 | 1.512933 | 1.927281 | 1.058589 | 1.422236 |
| **PLI** | 0.752266 | 0.876776 | 0.823871 | 0.786171 | 1.167153 | 0.795081 | 0.866886 |
| **SP** | SP1 | SP2 | SP3 | SP4 | SP5 | SP6 | Mean |
| As | 0.381452 | 0.677224 | 0.656551 | 0.449129 | 0.332102 | 0.806215 | 0.550446 |
| Ba | 0.114331 | 0.125883 | 0.118076 | 0.117638 | 0.105739 | 0.147849 | 0.121586 |
| Cd | 0.126341 | 0.150488 | 0.509122 | 1.128415 | 0.120122 | 0.256878 | 0.381894 |
| Co | 0.509571 | 0.633894 | 0.819282 | 1.300154 | 0.476159 | 0.743724 | 0.747131 |
| Cr | 1.529009 | 1.232690 | 1.664584 | 1.575826 | 1.552176 | 1.233813 | 1.464683 |
| Cu | 0.782572 | 0.749498 | 0.57566 | 0.557253 | 1.079796 | 0.726678 | 0.745243 |
| Mn | 0.580659 | 0.645059 | 0.638865 | 0.649915 | 0.658499 | 0.773782 | 0.657796 |
| Ni | 0.909242 | 0.825737 | 1.381238 | 1.852459 | 1.018900 | 0.904527 | 1.148684 |
| Pb | 0.271255 | 1.399983 | 0.416213 | 0.651639 | 0.368163 | 0.593314 | 0.616761 |
| Sb | 0.715522 | 1.729358 | 0.606567 | 0.859149 | 0.751836 | 1.108478 | 0.961818 |
| Zn | 2.369789 | 1.451495 | 0.718633 | 0.751466 | 2.850405 | 1.685641 | 1.637905 |
| **PLI** | 0.523885 | 0.67865 | 0.61731 | 0.736289 | 0.56245 | 0.686036 | 0.634103 |
| **MH** | SP1 | SP2 | SP3 | SP4 | SP5 | SP6 | Mean |
| As | 0.904895 | 0.592387 | 0.890883 | 0.745884 | 0.881378 | 1.046269 | 0.843616 |
| Ba | 0.142186 | 0.156380 | 0.215054 | 0.24536 | 0.250125 | 0.258151 | 0.211209 |
| Cd | 0.130049 | 0.109244 | 0.315927 | 0.130293 | 0.196244 | 0.16061 | 0.173728 |
| Co | 0.806331 | 1.143242 | 0.957681 | 0.956338 | 1.120839 | 1.119196 | 1.017271 |
| Cr | 1.643434 | 1.642335 | 1.938628 | 1.464030 | 1.622620 | 1.672028 | 1.663846 |
| Cu | 1.214266 | 0.661461 | 1.089425 | 0.617731 | 0.60825 | 0.974024 | 0.86086 |
| Mn | 0.993531 | 0.865606 | 0.920513 | 1.317756 | 1.279456 | 1.677781 | 1.175774 |
| Ni | 0.869499 | 1.610139 | 1.083920 | 1.028799 | 0.943265 | 0.980378 | 1.086000 |
| Pb | 0.600049 | 0.605703 | 6.336570 | 0.864879 | 0.801300 | 0.978579 | 1.697847 |
| Sb | 1.122701 | 1.204269 | 5.049687 | 0.975776 | 0.904507 | 1.514821 | 1.795294 |
| Zn | 1.196777 | 1.077203 | 1.892985 | 1.001263 | 1.154332 | 1.217352 | 1.256652 |
| **PLI** | 0.696634 | 0.677323 | 1.199539 | 0.711418 | 0.759238 | 0.876544 | 0.820116 |

Table S7. Ecological risk (Er) and ecological risk index (RI) for each sampling point across sites. SP1‒SP6, sampling points.

|  | Er | | | | | | | | | **RI** |
| --- | --- | --- | --- | --- | --- | --- | --- | --- | --- | --- |
| **TS** | As | Cd | Co | Cr | Cu | Mn | Ni | Pb | Zn |  |
| SP1 | 13.93 | 6.91 | 8.38 | 6.13 | 5.24 | 1.76 | 15.60 | 6.11 | 2.97 | 67.03 |
| SP2 | 12.61 | 7.01 | 9.01 | 5.30 | 5.23 | 1.90 | 9.54 | 6.15 | 2.55 | 59.30 |
| SP3 | 15.95 | 6.19 | 8.66 | 4.88 | 3.70 | 2.31 | 6.95 | 6.91 | 1.00 | 56.56 |
| SP4 | 17.83 | 5.44 | 9.36 | 5.54 | 4.88 | 1.60 | 8.42 | 5.02 | 1.96 | 60.04 |
| SP5 | 9.58 | 7.41 | 9.54 | 5.28 | 6.05 | 2.38 | 8.16 | 6.25 | 2.22 | 56.86 |
| SP6 | 9.79 | 6.30 | 7.04 | 4.59 | 5.14 | 1.69 | 7.54 | 4.93 | 2.61 | 49.64 |
| **LS** |  |  |  |  |  |  |  |  |  |  |
| SP1 | 9.20 | 6.72 | 7.52 | 4.59 | 3.05 | 1.56 | 6.06 | 5.55 | 0.88 | 45.13 |
| SP2 | 8.32 | 10.64 | 4.93 | 4.30 | 4.07 | 1.08 | 4.72 | 7.67 | 2.47 | 48.20 |
| SP3 | 6.81 | 25.64 | 4.82 | 3.20 | 3.12 | 1.14 | 10.41 | 3.92 | 1.00 | 60.05 |
| SP4 | 9.39 | 13.25 | 8.12 | 5.15 | 5.43 | 1.60 | 7.19 | 16.70 | 2.48 | 69.32 |
| SP5 | 8.59 | 5.14 | 5.93 | 4.01 | 2.79 | 1.34 | 4.87 | 5.65 | 1.07 | 39.39 |
| SP6 | 14.10 | 6.50 | 5.24 | 3.44 | 5.31 | 1.20 | 4.56 | 5.72 | 1.06 | 47.11 |
| **NG1** |  |  |  |  |  |  |  |  |  |  |
| SP1 | 5.31 | 4.48 | 4.82 | 4.38 | 3.40 | 1.20 | 3.64 | 6.56 | 0.83 | 34.62 |
| SP2 | 7.44 | 5.58 | 4.91 | 3.84 | 4.09 | 1.22 | 4.35 | 6.36 | 0.94 | 38.74 |
| SP3 | 4.53 | 5.84 | 4.89 | 3.00 | 4.25 | 0.92 | 5.85 | 4.79 | 2.80 | 36.87 |
| SP4 | 7.12 | 8.55 | 5.67 | 3.23 | 4.44 | 1.13 | 5.54 | 9.02 | 1.22 | 45.92 |
| SP5 | 8.14 | 9.43 | 4.92 | 3.15 | 4.64 | 1.09 | 5.28 | 6.03 | 1.26 | 43.95 |
| SP6 | 9.49 | 9.51 | 5.67 | 4.05 | 5.08 | 1.43 | 6.61 | 9.32 | 2.41 | 53.56 |
| **NG2** |  |  |  |  |  |  |  |  |  |  |
| SP1 | 8.83 | 5.94 | 6.33 | 4.15 | 3.69 | 1.52 | 7.09 | 4.68 | 1.63 | 43.86 |
| SP2 | 9.49 | 7.71 | 6.92 | 4.20 | 3.80 | 1.20 | 7.27 | 5.33 | 1.56 | 47.48 |
| SP3 | 5.22 | 4.38 | 6.77 | 3.80 | 2.91 | 1.15 | 6.20 | 3.40 | 0.84 | 34.69 |
| SP4 | 8.07 | 8.68 | 7.40 | 4.33 | 3.84 | 1.50 | 7.32 | 12.51 | 1.45 | 55.09 |
| SP5 | 7.80 | 12.29 | 5.44 | 3.92 | 3.75 | 1.07 | 6.42 | 7.01 | 1.29 | 48.99 |
| SP6 | 6.24 | 9.24 | 10.07 | 3.92 | 4.38 | 2.63 | 12.32 | 11.82 | 1.11 | 61.72 |
| **PN** |  |  |  |  |  |  |  |  |  |  |
| SP1 | 6.71 | 4.23 | 4.88 | 3.05 | 3.31 | 1.09 | 8.03 | 4.35 | 1.29 | 36.94 |
| SP2 | 6.57 | 23.06 | 4.83 | 3.54 | 3.58 | 0.97 | 8.03 | 4.57 | 1.44 | 56.58 |
| SP3 | 8.83 | 3.86 | 4.98 | 3.63 | 3.41 | 1.24 | 8.00 | 3.98 | 1.30 | 39.24 |
| SP4 | 6.31 | 4.82 | 4.72 | 3.52 | 3.50 | 1.07 | 7.24 | 3.64 | 1.51 | 36.33 |
| SP5 | 9.44 | 8.62 | 6.01 | 3.22 | 15.54 | 1.69 | 5.82 | 6.76 | 1.93 | 59.03 |
| SP6 | 8.24 | 4.38 | 4.97 | 2.95 | 3.16 | 1.11 | 5.41 | 4.15 | 1.06 | 35.43 |
| **SP** |  |  |  |  |  |  |  |  |  |  |
| SP1 | 3.81 | 3.79 | 2.55 | 3.06 | 3.91 | 0.58 | 4.55 | 1.36 | 2.37 | 25.98 |
| SP2 | 6.77 | 4.51 | 3.17 | 2.47 | 3.75 | 0.65 | 4.13 | 7.00 | 1.45 | 33.89 |
| SP3 | 6.57 | 15.27 | 4.10 | 3.33 | 2.88 | 0.64 | 6.91 | 2.08 | 0.72 | 42.49 |
| SP4 | 4.49 | 33.85 | 6.50 | 3.15 | 2.79 | 0.65 | 9.26 | 3.26 | 0.75 | 64.70 |
| SP5 | 3.32 | 3.60 | 2.38 | 3.10 | 5.40 | 0.66 | 5.09 | 1.84 | 2.85 | 28.25 |
| SP6 | 8.06 | 7.71 | 3.72 | 2.47 | 3.63 | 0.77 | 4.52 | 2.97 | 1.69 | 35.54 |
| **MH** |  |  |  |  |  |  |  |  |  |  |
| SP1 | 9.05 | 3.90 | 4.03 | 3.29 | 6.07 | 0.99 | 4.35 | 3.00 | 1.20 | 35.88 |
| SP2 | 5.92 | 3.28 | 5.72 | 3.28 | 3.31 | 0.87 | 8.05 | 3.03 | 1.08 | 34.53 |
| SP3 | 8.91 | 9.48 | 4.79 | 3.88 | 5.45 | 0.92 | 5.42 | 31.68 | 1.89 | 72.42 |
| SP4 | 7.46 | 3.91 | 4.78 | 2.93 | 3.09 | 1.32 | 5.14 | 4.32 | 1.00 | 33.95 |
| SP5 | 8.81 | 5.89 | 5.60 | 3.25 | 3.04 | 1.28 | 4.72 | 4.01 | 1.15 | 37.75 |
| SP6 | 10.46 | 4.82 | 5.60 | 3.34 | 4.87 | 1.68 | 4.90 | 4.89 | 1.22 | 41.78 |

Table S8. Mean Hazard Quotient (HQ) and Hazard Index (HI) associated with ingestion of soil trace elements. n = 6.

| Trace elements | TS |  | LS |  | NG1 |  | NG2 |  | PN |  | SP |  | MH |  |
| --- | --- | --- | --- | --- | --- | --- | --- | --- | --- | --- | --- | --- | --- | --- |
|  | Adult | Children | Adult | Children | Adult | Children | Adult | Children | Adult | Children | Adult | Children | Adult | Children |
| As | 0.0414 | 0.3866 | 0.0293 | 0.2737 | 0.0237 | 0.2215 | 0.0218 | 0.2039 | 0.0240 | 0.2236 | 0.0172 | 0.1602 | 0.0263 | 0.2456 |
| Ba | 0.0013 | 0.0118 | 0.0008 | 0.0078 | 0.0010 | 0.0092 | 0.0007 | 0.0067 | 0.0008 | 0.0072 | 0.0004 | 0.0036 | 0.0007 | 0.0062 |
| Cd | 0.0001 | 0.0011 | 0.0002 | 0.0020 | 0.0002 | 0.0014 | 0.0001 | 0.0013 | 0.0002 | 0.0014 | 0.0002 | 0.0020 | 0.0001 | 0.0009 |
| Co | 0.0009 | 0.0083 | 0.0006 | 0.0059 | 0.0007 | 0.0069 | 0.0005 | 0.0050 | 0.0005 | 0.0049 | 0.0004 | 0.0036 | 0.0005 | 0.0049 |
| Cr | 0.0001 | 0.0013 | 0.0001 | 0.0010 | 0.0001 | 0.0010 | 0.0001 | 0.0009 | 0.0001 | 0.0008 | 0.0001 | 0.0007 | 0.0001 | 0.0008 |
| Cu | 0.0013 | 0.0125 | 0.0011 | 0.0099 | 0.0010 | 0.0093 | 0.0012 | 0.0107 | 0.0014 | 0.0135 | 0.0010 | 0.0093 | 0.0011 | 0.0107 |
| Mn | 0.0093 | 0.0864 | 0.0063 | 0.0588 | 0.0072 | 0.0673 | 0.0056 | 0.0519 | 0.0057 | 0.0532 | 0.0031 | 0.0293 | 0.0056 | 0.0524 |
| Ni | 0.0037 | 0.0347 | 0.0025 | 0.0234 | 0.0031 | 0.0288 | 0.0021 | 0.0193 | 0.0028 | 0.0263 | 0.0023 | 0.0213 | 0.0022 | 0.0201 |
| Pb | 0.0125 | 0.1162 | 0.0159 | 0.1486 | 0.0158 | 0.1471 | 0.0148 | 0.1384 | 0.0097 | 0.0902 | 0.0065 | 0.0608 | 0.0179 | 0.1675 |
| Sb | 0.0061 | 0.0570 | 0.0032 | 0.0295 | 0.0035 | 0.0329 | 0.0031 | 0.0294 | 0.0039 | 0.0363 | 0.0022 | 0.0206 | 0.0041 | 0.0384 |
| Zn | 0.0007 | 0.0066 | 0.0005 | 0.0044 | 0.0004 | 0.0039 | 0.0005 | 0.0047 | 0.0005 | 0.0042 | 0.0005 | 0.0049 | 0.0004 | 0.0037 |
| HI | 0.0774 | 0.7229 | 0.0605 | 0.5649 | 0.0567 | 0.5293 | 0.0506 | 0.4722 | 0.0495 | 0.4616 | 0.0339 | 0.3163 | 0.0591 | 0.5513 |

Table S9. Mean Hazard Quotient (HQ) and Hazard Index (HI) related to dermal contact with soil trace elements. n = 6.

| Trace elements | TS |  | LS |  | NG1 |  | NG2 |  | PN |  | SP |  | MH |  |
| --- | --- | --- | --- | --- | --- | --- | --- | --- | --- | --- | --- | --- | --- | --- |
|  | Adult | Children | Adult | Children | Adult | Children | Adult | Children | Adult | Children | Adult | Children | Adult | Children |
| As | 0.0121 | 0.0792 | 0.0086 | 0.0561 | 0.0069 | 0.0454 | 0.0064 | 0.0418 | 0.0070 | 0.0458 | 0.0050 | 0.0328 | 0.0077 | 0.0503 |
| Ba | 0.0000 | 0.0001 | 0.0000 | 0.0000 | 0.0000 | 0.0001 | 0.0000 | 0.0000 | 0.0000 | 0.0000 | 0.0000 | 0.0000 | 0.0000 | 0.0000 |
| Cd | 0.0015 | 0.0096 | 0.0025 | 0.0166 | 0.0018 | 0.0118 | 0.0016 | 0.0106 | 0.0018 | 0.0120 | 0.0026 | 0.0168 | 0.0012 | 0.0076 |
| Co | 0.0002 | 0.0013 | 0.0001 | 0.0009 | 0.0002 | 0.0011 | 0.0001 | 0.0008 | 0.0001 | 0.0008 | 0.0001 | 0.0006 | 0.0001 | 0.0008 |
| Cr | 0.0013 | 0.0087 | 0.0010 | 0.0067 | 0.0010 | 0.0066 | 0.0009 | 0.0059 | 0.0008 | 0.0054 | 0.0007 | 0.0048 | 0.0008 | 0.0055 |
| Cu | 0.0005 | 0.0035 | 0.0004 | 0.0028 | 0.0004 | 0.0026 | 0.0005 | 0.0030 | 0.0006 | 0.0038 | 0.0004 | 0.0026 | 0.0005 | 0.0030 |
| Mn | 0.0843 | 0.5524 | 0.0574 | 0.3757 | 0.0657 | 0.4302 | 0.0507 | 0.3318 | 0.0519 | 0.3402 | 0.0286 | 0.1874 | 0.0511 | 0.3349 |
| Ni | 0.0016 | 0.0108 | 0.0011 | 0.0073 | 0.0014 | 0.0090 | 0.0009 | 0.0060 | 0.0012 | 0.0082 | 0.0010 | 0.0066 | 0.0010 | 0.0063 |
| Pb | 0.0010 | 0.0065 | 0.0013 | 0.0083 | 0.0013 | 0.0082 | 0.0012 | 0.0077 | 0.0008 | 0.0051 | 0.0005 | 0.0034 | 0.0014 | 0.0094 |
| Sb | 0.0001 | 0.0007 | 0.0001 | 0.0004 | 0.0001 | 0.0004 | 0.0001 | 0.0004 | 0.0001 | 0.0005 | 0.0000 | 0.0003 | 0.0001 | 0.0005 |
| Zn | 0.0004 | 0.0028 | 0.0003 | 0.0019 | 0.0003 | 0.0016 | 0.0003 | 0.0020 | 0.0003 | 0.0018 | 0.0003 | 0.0021 | 0.0002 | 0.0016 |
| HI | 0.1032 | 0.6756 | 0.0728 | 0.4766 | 0.0789 | 0.5170 | 0.0626 | 0.4100 | 0.0647 | 0.4235 | 0.0393 | 0.2573 | 0.0641 | 0.4198 |

Table S10. Cancer risk (CR) through ingestion (CRO) and dermal contact (CRD) and total cancer risk (TCR) from assessed soil trace elements. SP1‒SP6, sampling points.

| As |  |  |  |  |  |  |  |
| --- | --- | --- | --- | --- | --- | --- | --- |
| Adults |  | SP1 | SP2 | SP3 | SP4 | SP5 | SP6 |
| TS | CRO | 1.95E-05 | 1.77E-05 | 2.24E-05 | 2.5E-05 | 1.34E-05 | 1.37E-05 |
|  | CRD | 5.71E-06 | 5.17E-06 | 6.54E-06 | 7.31E-06 | 3.93E-06 | 4.01E-06 |
|  | TCR | 2.53E-05 | 2.29E-05 | 2.89E-05 | 3.23E-05 | 1.74E-05 | 1.78E-05 |
| LS | CRO | 1.29E-05 | 1.17E-05 | 9.56E-06 | 1.32E-05 | 1.21E-05 | 1.98E-05 |
|  | CRD | 3.77E-06 | 3.41E-06 | 2.79E-06 | 3.85E-06 | 3.52E-06 | 5.78E-06 |
|  | TCR | 1.67E-05 | 1.51E-05 | 1.24E-05 | 1.7E-05 | 1.56E-05 | 2.56E-05 |
| NG1 | CRO | 1.24E-05 | 1.33E-05 | 7.33E-06 | 1.13E-05 | 1.09E-05 | 8.76E-06 |
|  | CRD | 2.18E-06 | 3.05E-06 | 1.86E-06 | 2.92E-06 | 3.34E-06 | 3.89E-06 |
|  | TCR | 1.46E-05 | 1.64E-05 | 9.19E-06 | 1.42E-05 | 1.43E-05 | 1.26E-05 |
| NG2 | CRO | 7.46E-06 | 1.04E-05 | 6.36E-06 | 9.99E-06 | 1.14E-05 | 1.33E-05 |
|  | CRD | 3.62E-06 | 3.89E-06 | 2.14E-06 | 3.31E-06 | 3.2E-06 | 2.56E-06 |
|  | TCR | 1.11E-05 | 1.43E-05 | 8.5E-06 | 1.33E-05 | 1.46E-05 | 1.59E-05 |
| PN | CRO | 9.41E-06 | 9.22E-06 | 1.24E-05 | 8.85E-06 | 1.32E-05 | 1.16E-05 |
|  | CRD | 2.75E-06 | 2.69E-06 | 3.62E-06 | 2.58E-06 | 3.87E-06 | 3.38E-06 |
|  | TCR | 1.22E-05 | 1.19E-05 | 1.6E-05 | 1.14E-05 | 1.71E-05 | 1.49E-05 |
| SP | CRO | 5.35E-06 | 9.5E-06 | 9.21E-06 | 6.3E-06 | 4.66E-06 | 1.13E-05 |
|  | CRD | 1.56E-06 | 2.78E-06 | 2.69E-06 | 1.84E-06 | 1.36E-06 | 3.3E-06 |
|  | TCR | 6.92E-06 | 1.23E-05 | 1.19E-05 | 8.14E-06 | 6.02E-06 | 1.46E-05 |
| MH | CRO | 1.27E-05 | 8.31E-06 | 1.25E-05 | 1.05E-05 | 1.24E-05 | 1.47E-05 |
|  | CRD | 3.71E-06 | 2.43E-06 | 3.65E-06 | 3.06E-06 | 3.61E-06 | 4.29E-06 |
|  | TCR | 1.64E-05 | 1.07E-05 | 1.62E-05 | 1.35E-05 | 1.6E-05 | 1.9E-05 |
| Children |  | SP1 | SP2 | SP3 | SP4 | SP5 | SP6 |
| TS | CRO | 1.82E-04 | 1.65E-04 | 2.09E-04 | 2.34E-04 | 1.25E-04 | 1.28E-04 |
|  | CRD | 3.74E-05 | 3.38E-05 | 4.28E-05 | 4.79E-05 | 2.57E-05 | 2.63E-05 |
|  | TCR | 2.20E-04 | 1.99E-04 | 2.52E-04 | 2.81E-04 | 1.51E-04 | 1.55E-04 |
| LS | CRO | 1.21E-04 | 1.09E-04 | 8.93E-05 | 1.23E-04 | 1.12E-04 | 1.85E-04 |
|  | CRD | 2.47E-05 | 2.23E-05 | 1.83E-05 | 2.52E-05 | 2.31E-05 | 3.78E-05 |
|  | TCR | 1.45E-04 | 1.31E-04 | 1.08E-04 | 1.48E-04 | 1.36E-04 | 2.22E-04 |
| NG1 | CRO | 1.16E-04 | 1.24E-04 | 6.84E-05 | 1.06E-04 | 1.02E-04 | 8.17E-05 |
|  | CRD | 1.43E-05 | 2.00E-05 | 1.22E-05 | 1.91E-05 | 2.19E-05 | 2.55E-05 |
|  | TCR | 1.30E-04 | 1.44E-04 | 8.06E-05 | 1.25E-04 | 1.24E-04 | 1.07E-04 |
| NG2 | CRO | 6.96E-05 | 9.74E-05 | 5.93E-05 | 9.32E-05 | 1.07E-04 | 1.24E-04 |
|  | CRD | 2.37E-05 | 2.55E-05 | 1.40E-05 | 2.17E-05 | 2.09E-05 | 1.68E-05 |
|  | TCR | 9.33E-05 | 1.23E-04 | 7.34E-05 | 1.15E-04 | 1.28E-04 | 1.41E-04 |
| PN | CRO | 8.78E-05 | 8.60E-05 | 1.16E-04 | 8.26E-05 | 1.24E-04 | 1.08E-04 |
|  | CRD | 1.80E-05 | 1.76E-05 | 2.37E-05 | 1.69E-05 | 2.53E-05 | 2.21E-05 |
|  | TCR | 1.06E-04 | 1.04E-04 | 1.39E-04 | 9.95E-05 | 1.49E-04 | 1.30E-04 |
| SP | CRO | 5.00E-05 | 8.87E-05 | 8.60E-05 | 5.88E-05 | 4.35E-05 | 1.06E-04 |
|  | CRD | 1.02E-05 | 1.82E-05 | 1.76E-05 | 1.21E-05 | 8.92E-06 | 2.16E-05 |
|  | TCR | 6.02E-05 | 1.07E-04 | 1.04E-04 | 7.09E-05 | 5.24E-05 | 1.27E-04 |
| MH | CRO | 1.19E-04 | 7.76E-05 | 1.17E-04 | 9.77E-05 | 1.15E-04 | 1.37E-04 |
|  | CRD | 2.43E-05 | 1.59E-05 | 2.39E-05 | 2.00E-05 | 2.37E-05 | 2.81E-05 |
|  | TCR | 1.43E-04 | 9.35E-05 | 1.41E-04 | 1.18E-04 | 1.39E-04 | 1.65E-04 |
| Cd |  |  |  |  |  |  |  |
| Adults |  | SP1 | SP2 | SP3 | SP4 | SP5 | SP6 |
| TS | CRO | 7.9E-07 | 8.01E-07 | 7.07E-07 | 6.21E-07 | 8.46E-07 | 7.19E-07 |
|  | CRD | 9.45E-08 | 9.59E-08 | 8.46E-08 | 7.43E-08 | 1.01E-07 | 8.61E-08 |
|  | TCR | 8.84E-07 | 8.97E-07 | 7.92E-07 | 6.95E-07 | 9.47E-07 | 8.05E-07 |
| LS | CRO | 7.68E-07 | 1.22E-06 | 2.93E-06 | 1.51E-06 | 5.87E-07 | 7.42E-07 |
|  | CRD | 9.19E-08 | 1.45E-07 | 3.51E-07 | 1.81E-07 | 7.03E-08 | 8.88E-08 |
|  | TCR | 8.6E-07 | 1.36E-06 | 3.28E-06 | 1.69E-06 | 6.57E-07 | 8.31E-07 |
| NG1 | CRO | 6.79E-07 | 8.81E-07 | 5E-07 | 9.91E-07 | 1.4E-06 | 1.05E-06 |
|  | CRD | 6.12E-08 | 7.63E-08 | 7.98E-08 | 1.17E-07 | 1.29E-07 | 1.3E-07 |
|  | TCR | 7.4E-07 | 9.57E-07 | 5.8E-07 | 1.11E-06 | 1.53E-06 | 1.18E-06 |
| NG2 | CRO | 5.11E-07 | 6.37E-07 | 6.67E-07 | 9.76E-07 | 1.08E-06 | 1.09E-06 |
|  | CRD | 8.12E-08 | 1.05E-07 | 5.99E-08 | 1.19E-07 | 1.68E-07 | 1.26E-07 |
|  | TCR | 5.92E-07 | 7.43E-07 | 7.27E-07 | 1.09E-06 | 1.25E-06 | 1.21E-06 |
| PN | CR_O_ | 4.83E-07 | 2.63E-06 | 4.41E-07 | 5.5E-07 | 9.84E-07 | 5E-07 |
|  | CRD | 5.78E-08 | 3.15E-07 | 5.28E-08 | 6.59E-08 | 1.18E-07 | 5.99E-08 |
|  | TCR | 5.4E-07 | 2.95E-06 | 4.94E-07 | 6.16E-07 | 1.1E-06 | 5.6E-07 |
| SP | CRO | 4.33E-07 | 5.16E-07 | 1.74E-06 | 3.87E-06 | 4.12E-07 | 8.8E-07 |
|  | CRD | 5.18E-08 | 6.17E-08 | 2.09E-07 | 4.63E-07 | 4.93E-08 | 1.05E-07 |
|  | TCR | 4.85E-07 | 5.77E-07 | 1.95E-06 | 4.33E-06 | 4.61E-07 | 9.85E-07 |
| MH | CRO | 4.46E-07 | 3.74E-07 | 1.08E-06 | 4.46E-07 | 6.72E-07 | 5.5E-07 |
|  | CRD | 5.33E-08 | 4.48E-08 | 1.3E-07 | 5.34E-08 | 8.05E-08 | 6.59E-08 |
|  | TCR | 4.99E-07 | 4.19E-07 | 1.21E-06 | 5E-07 | 7.53E-07 | 6.16E-07 |
| Children |  | SP1 | SP2 | SP3 | SP4 | SP5 | SP6 |
| TS | CRO | 7.37E-06 | 7.47E-06 | 6.6E-06 | 5.8E-06 | 7.9E-06 | 6.71E-06 |
|  | CRD | 6.19E-07 | 6.28E-07 | 5.54E-07 | 4.87E-07 | 6.63E-07 | 5.64E-07 |
|  | TCR | 7.99E-06 | 8.1E-06 | 7.15E-06 | 6.28E-06 | 8.56E-06 | 7.28E-06 |
| LS | CRO | 7.17E-06 | 1.13E-05 | 2.73E-05 | 1.41E-05 | 5.48E-06 | 6.92E-06 |
|  | CRD | 6.02E-07 | 9.53E-07 | 2.3E-06 | 1.19E-06 | 4.6E-07 | 5.82E-07 |
|  | TCR | 7.77E-06 | 1.23E-05 | 2.96E-05 | 1.53E-05 | 5.94E-06 | 7.5E-06 |
| NG1 | CRO | 6.34E-06 | 8.22E-06 | 4.67E-06 | 9.25E-06 | 1.31E-05 | 9.84E-06 |
|  | CRD | 4.01E-07 | 5E-07 | 5.23E-07 | 7.65E-07 | 8.45E-07 | 8.52E-07 |
|  | TCR | 6.74E-06 | 8.72E-06 | 5.19E-06 | 1E-05 | 1.39E-05 | 1.07E-05 |
| NG2 | CRO | 4.77E-06 | 5.95E-06 | 6.22E-06 | 9.11E-06 | 1.01E-05 | 1.01E-05 |
|  | CRD | 5.32E-07 | 6.9E-07 | 3.92E-07 | 7.77E-07 | 1.1E-06 | 8.27E-07 |
|  | TCR | 5.3E-06 | 6.64E-06 | 6.61E-06 | 9.89E-06 | 1.12E-05 | 1.1E-05 |
| PN | CRO | 4.5E-06 | 2.46E-05 | 4.12E-06 | 5.14E-06 | 9.18E-06 | 4.67E-06 |
|  | CRD | 3.78E-07 | 2.07E-06 | 3.46E-07 | 4.32E-07 | 7.71E-07 | 3.92E-07 |
|  | TCR | 4.88E-06 | 2.66E-05 | 4.46E-06 | 5.57E-06 | 9.95E-06 | 5.06E-06 |
| SP | CRO | 4.04E-06 | 4.81E-06 | 1.63E-05 | 3.61E-05 | 3.84E-06 | 8.21E-06 |
|  | CRD | 3.39E-07 | 4.04E-07 | 1.37E-06 | 3.03E-06 | 3.23E-07 | 6.9E-07 |
|  | TCR | 4.38E-06 | 5.22E-06 | 1.76E-05 | 3.91E-05 | 4.16E-06 | 8.9E-06 |
| MH | CRO | 4.16E-06 | 3.49E-06 | 1.01E-05 | 4.17E-06 | 6.28E-06 | 5.14E-06 |
|  | CRD | 3.49E-07 | 2.93E-07 | 8.49E-07 | 3.5E-07 | 5.27E-07 | 4.31E-07 |
|  | TCR | 4.51E-06 | 3.79E-06 | 1.1E-05 | 4.52E-06 | 6.8E-06 | 5.57E-06 |
| Pb (Ingestion) |  | SP1 | SP2 | SP3 | SP4 | SP5 | SP6 |
| TS | Adults | 3.84E-07 | 3.87E-07 | 4.34E-07 | 3.16E-07 | 3.93E-07 | 3.1E-07 |
|  | Children | 3.58E-06 | 3.61E-06 | 4.05E-06 | 2.95E-06 | 3.67E-06 | 2.89E-06 |
| LS | Adults | 3.49E-07 | 4.82E-07 | 2.47E-07 | 1.05E-06 | 3.55E-07 | 3.6E-07 |
|  | Children | 3.26E-06 | 4.5E-06 | 2.3E-06 | 9.8E-06 | 3.32E-06 | 3.36E-06 |
| NG1 | Adults | 3.84E-07 | 3.87E-07 | 4.34E-07 | 3.16E-07 | 3.93E-07 | 3.1E-07 |
|  | Children | 3.58E-06 | 3.61E-06 | 4.05E-06 | 2.95E-06 | 3.67E-06 | 2.89E-06 |
| NG2 | Adults | 2.94E-07 | 3.35E-07 | 2.14E-07 | 7.87E-07 | 4.41E-07 | 7.43E-07 |
|  | Children | 2.75E-06 | 3.13E-06 | 2E-06 | 7.34E-06 | 4.11E-06 | 6.94E-06 |
| PN | Adults | 2.74E-07 | 2.87E-07 | 2.5E-07 | 2.29E-07 | 4.25E-07 | 2.61E-07 |
|  | Children | 2.55E-06 | 2.68E-06 | 2.34E-06 | 2.14E-06 | 3.96E-06 | 2.43E-06 |
| SP | Adults | 8.53E-08 | 4.4E-07 | 1.31E-07 | 2.05E-07 | 1.16E-07 | 1.87E-07 |
|  | Children | 7.96E-07 | 4.11E-06 | 1.22E-06 | 1.91E-06 | 1.08E-06 | 1.74E-06 |
| MH | Adults | 1.89E-07 | 1.9E-07 | 1.99E-06 | 2.72E-07 | 2.52E-07 | 3.08E-07 |
|  | Children | 1.76E-06 | 1.78E-06 | 1.86E-05 | 2.54E-06 | 2.35E-06 | 2.87E-06 |

**References**

DEA (2010) Department of Environmental Affairs, The framework for the management of contaminated land, South Africa. <https://cer.org.za/wp-content/uploads/2012/06/Framework-for-the-Management-of-Contaminated-Land-May-2010.pdf>

Finley BL, Monnot AD, Paustenbach DJ, Gaffney SH (2012) Derivation of a chronic oral reference dose for cobalt. Regul Toxicol Pharmacol 64:491–503. <https://doi.org/10.1016/j.yrtph.2012.08.022>

Håkanson L (1980) An ecological risk index for aquatic pollution control. A sedimentological approach. Water Res 14(8):975–1001. <https://doi.org/10.1016/0043-1354(80)90143-8>

IRIS (1989) Integrated Risk Information System, Chemical Assessment Summary: Cadmium; CASRN 7440-43-9; U.S. Environmental Protection Agency, National Center for Environmental Assessment. Retrieved January 7, 2025, from <https://iris.epa.gov/static/pdfs/0141_summary.pdf>

IRIS (1991) Integrated Risk Information System, Arsenic, Inorganic CASRN 7440-38-2; U.S. Environmental Protection Agency, National Center for Environmental Assessment. Retrieved

January 7, 2025, from <https://iris.epa.gov/static/pdfs/0278_summary.pdf>

IRIS (2004) Integrated Risk Information System, Chemical Assessment Summary: Lead and compounds (inorganic); CASRN 7439-92-1; U.S. Environmental Protection Agency, National Center for Environmental Assessment. Retrieved January 7, 2025, from <https://cfpub.epa.gov/ncea/iris/iris_documents/documents/subst/0277_summary.pdf>

IRIS (2005) Integrated Risk Information System, Chemical Assessment Summary: Barium and compounds; CASRN 7440-39-3; U.S. Environmental Protection Agency, National Center for Environmental Assessment. Retrieved January 7, 2025, from <https://iris.epa.gov/static/pdfs/0010_summary.pdf>

Jiang HH, Cai LM, Hu GC, Wen HH, Luo J, Xu HQ, Chen LG (2021) An integrated exploration on health risk assessment quantification of potentially hazardous elements in soils from the perspective of sources. Ecotoxicol Environ Safety 208:111489. https://doi.org/10.1016/j.ecoenv.2020.111489

Miletić A, Lučić M, Onjia A (2023) Exposure factors in health risk assessment of heavy metal(loid)s in soil and sediment. Metals 13(7), 1266. <https://doi.org/10.3390/met13071266>

Müller G (1969) Index of geoaccumulation in sediments of the Rhine River. Geojournal 2:108–118.

Relić D, Sakan S, Anđelković I, Popović A, Đorđević D (2019) Pollution and health risk assessments of potentially toxic elements in soil and sediment samples in a petrochemical industry and surrounding area. Molecules 24(11), 2139. <https://doi.org/10.3390/molecules24112139>

Rinklebe J. Antoniadis V, Shaheen SM, Rosche O, Altermann M (2019) Health risk assessment of potentially toxic elements in soils along the Central Elbe River, Germany. Environ Int 126:76−88. <https://doi.org/10.1016/j.envint.2019.02.011>

Timofeev I, Kosheleva N, Kasimov N (2019) Health risk assessment based on the contents of potentially toxic elements in urban soils of Darkhan, Mongolia. J Environ Manage 242:279−289. <https://doi.org/10.1016/j.jenvman.2019.04.090>

US EPA (2002) United States Environmental Protection Agency, Supplemental Guidance for Developing Soil Screening Levels for Superfund Sites, Solid waste and emergency response. <https://nepis.epa.gov/Exe/ZyPDF.cgi/91003IJK.PDF?Dockey=91003IJK.PDF>
